# Spatial registration of neuron morphologies based on maximization of volume overlap

**DOI:** 10.1186/s12859-018-2136-z

**Published:** 2018-04-18

**Authors:** Ajayrama Kumaraswamy, Kazuki Kai, Hiroyuki Ai, Hidetoshi Ikeno, Thomas Wachtler

**Affiliations:** 1 Department of Biology II, Ludwig-Maximilians-Universität München, Grosshadernerstr, 2, Planegg-Martinsried, 82152 Germany; 2 Department of Earth System Science, Fukuoka University, 8-19-1 Nanakuma, Jonan-ku, Fukuoka-shi, Fukuoka, 814-0180 Japan; 3 School of Human Science and Environment, University of Hyogo, 1-1-12 Shinazaike-Honcho, Himeji, 670-0092 Hyogo Japan

**Keywords:** Spatial registration, Neuron morphology

## Abstract

**Background:**

Morphological features are widely used in the study of neuronal function and pathology. Invertebrate neurons are often structurally stereotypical, showing little variance in gross spatial features but larger variance in their fine features. Such variability can be quantified using detailed spatial analysis, which however requires the morphologies to be registered to a common frame of reference.

**Results:**

We outline here new algorithms — Reg-MaxS and Reg-MaxS-N — for co-registering pairs and groups of morphologies, respectively. Reg-MaxS applies a sequence of translation, rotation and scaling transformations, estimating at each step the transformation parameters that maximize spatial overlap between the volumes occupied by the morphologies. We test this algorithm with synthetic morphologies, showing that it can account for a wide range of transformation differences and is robust to noise. Reg-MaxS-N co-registers groups of more than two morphologies by iteratively calculating an average volume and registering all morphologies to this average using Reg-MaxS. We test Reg-MaxS-N using five groups of morphologies from the *Droshophila melanogaster* brain and identify the cases for which it outperforms existing algorithms and produce morphologies very similar to those obtained from registration to a standard brain atlas.

**Conclusions:**

We have described and tested algorithms for co-registering pairs and groups of neuron morphologies. We have demonstrated their application to spatial comparison of stereotypic morphologies and calculation of dendritic density profiles, showing how our algorithms for registering neuron morphologies can enable new approaches in comparative morphological analyses and visualization.

**Electronic supplementary material:**

The online version of this article (10.1186/s12859-018-2136-z) contains supplementary material, which is available to authorized users.

## Background

Since Ramon y Cajal’s ‘Neuron Theory’ [1], neuronal morphology has been a prominent field of study in Neuroscience. With early hand-drawn illustrations, later camera lucida tracings and more recent digital reconstructions [2], scientists have investigated the structure of individual nerve cells to better understand its role in neuronal function and pathology. Using modern imaging techniques and reconstruction algorithms, labs from around the world are producing huge numbers of detailed 3D morphologies [3, 4], and databases have been developed to collect and host such data [5].

A prominent application of neuron morphology is in comparative studies aiming to quantify the inter-group and intra-group variability of neurons. Neuronal shape and structure have been known to vary widely, even across specimens of a single species, making their characterization and classification a very difficult task [6]. Although long investigated [7, 8], the general principles underlying such diverse structures have largely been elusive, with a few widely applicable ones being uncovered only in the last decade [9–12]. Many different approaches with increasingly complex methods have therefore been used in the investigation of neuronal shape and structure.

A common approach has been to statistically test the variance of whole cell scalar measures ([13, 14]) of neuronal morphologies within and between groups. Although these methods have been successful in some cases [15–17], they have proven unsuitable for quantifying finer changes in topology and morphology [15, 18].

The next finer level of quantification involves dividing each morphology into concentric disks or shells about pre-identified centering points, grouping topologically or morphologically equidistant regions from different individuals and computing statistical variability of morphological and topological measures like the number of dendrites [19–21] within and across groups to characterize morphologies. For each such set of corresponding regions, statistical variability of morphological and topological measures like the number of dendrites [19–21] are used to characterize morphologies. Although this approach has been successfully used to quantify inter-group and intra-group variability in several studies of specific cell types [22–25], it has been found to be inadequate for morphologies that have similarly complex structures but differ in fine spatial distributions of morphological and topological features [15, 18]. For such cases, Mizrahi et al. [18] illustrated the use of Hausdorff Distance based features by quantifying the overall spatial dissimilarity between morphologies at different spatial scales. More recently, Kanari et al. [26] proposed a novel feature based on radial distance and topological “persistence” of dendrites and showed that a distance measure based on it could distinguish groups of complex morphologies with fine differences. A shortcoming of these approaches is that regions that are morphologically or topologically equidistant are lumped together for analysis, which can lead to dilution or cancellation of differences. Another drawback of this approach is the requirement for identification of corresponding centering points across different specimens, especially for invertebrates for which the somas are “segregated” [27] and variably located (for example, see Additional file 1 that visualizes classified groups of morphologies from *Drosophila melanogaster*).

For localization of inter-group and intra-group differences in morphological features, a spatial correspondence needs to be established between regions, in other words, the morphologies need to be co-aligned or co-registered. Several recent studies have proposed methods for such co-registration of morphologies and used them to compare morphologies.

Fiduciary markers can be used to register the original image data to a standard brain before extracting morphologies [28, 29]. Although this approach is very effective for brain regions with an existing standard brain [30–32], construction of a new standard brain is beyond the means of individual researchers as it requires a huge concerted effort. Furthermore, even for the cases where brain atlases are available, registration of individual morphologies can be ineffective due to lack of sufficient fiduciary markers in the brain region of interest. Hence methods that co-register morphologies without requiring external information are needed.

**Fig. 1:**
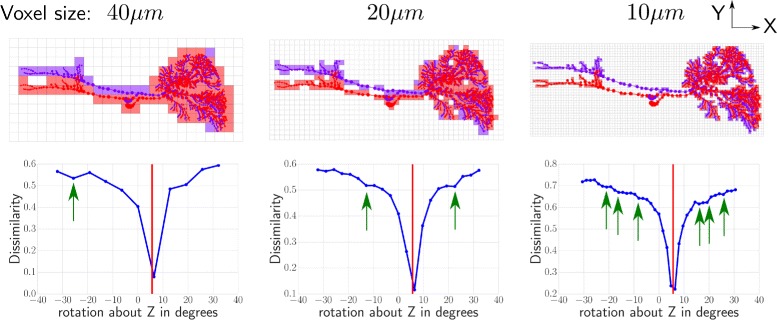
Volume representation of morphologies and spatial dissimilarity profiles at different voxel sizes illustrated using planar morphologies. Top row: Two example planar morphologies with volume representations at different voxel sizes. Circles visualize SWC points and lines their connectivity, with circle sizes indicating the diameter of the points. The two morphologies are identical but are rotated against each other about their centroids. Their discretized volume representations at corresponding voxel sizes are indicated by the filled squares. Bottom row: Variation of spatial dissimilarity between the morphologies at different voxel sizes as one of the morphologies (red morphology in top row) was rotated about its centroid. Dissimilarity was quantified using the spatial non-centric measure (see main text). The actual rotation difference between the morphologies is indicated by the red line. With decreasing voxel size, spatial dissimilarity profiles show increasing number of local minima (green arrows)

Other studies have presented co-registration methods that do not need fiduciary markers. Mizrahi et al. [18] implemented a method consisting of a translation for matching landmarks and rotation about one axis based on radii of ganglia. BlastNeuron [33] uses an affine registration method based on pointwise Euclidean distances and RANSAC sampling [34] as a preprocessing step for establishing detailed spatial and topological correspondence between morphologies. Several Iterative Closest Point (ICP) based methods from computer vision and biomedical imaging are also applicable, specifically the ones that can handle morphologies scaled differently along different axes [35, 36]. All these methods use measures of dissimilarity based on pointwise Euclidean distances for registration and hence seek a solution of point-to-point or surface-to-surface overlap, which can be hard to achieve for neuron morphologies, due to natural biological variation in their fine spatial structures. This has also been a major consideration in the construction and application of brain atlases [37]. Even neurons that have highly consistent global spatial features show considerable variation in their lower order branches [18, 37]. Moreover, the spatial region occupied by dendritic arbor has been shown to be important for the classification and synthesis of morphologies [15] and for investigating the role of single neuron morphology in the population [38]. This is consistent with dendrites and axons occupying specific spatial regions for making synaptic connections, while, within these regions, there can be variability in the exact arborization patterns at fine spatial scales [10]. Therefore, our approach aims to match the volume occupied by dendritic arbors at different spatial scales instead of seeking a point-to-point match between morphologies. Specifically, affine transformations are applied to blurred volume representations of morphologies at different spatial scales (Fig. 1) to maximize spatial overlap between volumes occupied by them. Using this approach, we present Reg-MaxS (**Reg**istration based on **Max**imization of **S**patial overlap) and Reg-MaxS-N for co-registering pairs and groups of morphologies, respectively.

## Methods

We describe here algorithms for co-registration of morphologies based on maximizing spatial overlap and such an approach requires defining a measure of spatial dissimilarity between morphologies and describing a strategy for finding a transformation that minimizes this dissimilarity. We discuss these aspects in the following subsections.

### Measures of spatial dissimilarity

Our algorithms approach spatial dissimilarity based on the overlap between volumes occupied by morphologies at different spatial scales. The following definition for volume occupied by morphologies is used.

#### Representing the volume of a morphology

A common way of representing a neuron’s three dimensional structure is by using the SWC format [14, 39], which represents a binary tree embedded in three dimensional space. Each point or node has, apart from its three spatial coordinates, a radius associated with it. With these features, every parent-child pair of nodes can be used to construct a frustrum, and consequently a set of connected frustra can be constructed from a tree structure which then represents the neuronal morphology. In our algorithms, to extract a volume representation of a morphology described in the SWC format, the three dimensional space containing the morphology is discretized into a set of equally sized cubic voxels (Fig. 1 top row). The voxels are positioned so that there is a voxel with its center at the origin of the space and the edge length of a voxel, which we term “voxel size”, is the most important parameter of this volume discretization. Among these voxels, those that contain at least one point of the morphology are identified and the resulting set of voxels is used to represent its volume.

#### Measures of spatial dissimilarity for two morphologies

Given two morphologies *A* and *B*, we define spatial dissimilarity (*D*) from their volume representations *setA* and *setB* as:

$$\begin{array}{*{20}l} D(setA,\ setB) &= \frac{n(setA\ -\ setB) + n(setB\ -\ setA)}{n(setA\ \cup \ setB)}\\ &= 1 - \frac{n(setA\ \cap \ setB)}{n(setA\ \cup \ setB)} \end{array} $$

where *n*() represents the number of elements in a set, and ∪ and ∩ represent the set union and set intersection operators, respectively. This measure essentially quantifies the spatial overlap between two morphologies normalized by their total volume.

Our algorithms use two measures of spatial dissimilarity, which we call “centric” and “non-centric” measures. The non-centric measure calculates the spatial dissimilarity between morphologies based on the values given, without applying any transformations. This measure is used when estimating translation and rotation differences between morphologies. The centric measure first translates one of the morphologies so that its centroid coincides with that of the other and calculates spatial dissimilarity using the volumes of the resulting morphologies. This measure is used when estimating scaling differences.

#### Measures of spatial dissimilarity for a group of morphologies

We define a measure for more than two morphologies based on voxel occupancy in the following paragraphs.

Given a group of morphologies, occupancy of a voxel is defined as the total number of morphologies of the group that have at least one point belonging to the voxel. A histogram of voxel occupancy values is calculated using all voxels with non-zero occupancy. A weighted histogram is created by multiplying each count of the histogram by its voxel occupancy. A normalized histogram is created by normalizing the weighted histogram by its sum.

It is desirable that a perfectly co-registered group of morphologies, i.e., a group with each morphology occupying the same set of voxels, has a spatial dissimilarity of zero. The normalized histogram of such a group would have a value of one at voxel occupancy equal to the size of the group and zero for all other values of voxel occupancy. Larger deviation from such a normalized histogram indicates larger spatial dissimilarity. Therefore, we define spatial dissimilarity of a group of morphologies as the distance between its normalized histogram and the normalized histogram corresponding to perfect spatial overlap, quantified by Earth-Mover-Distance [40].

### Estimating best transformations

In our approach, morphologies are co-registered by repeatedly removing rotation, scaling or translation differences. These differences are estimated using a multi-scale method based on exhaustive searches, which are described in the following paragraphs. Since the measures defined above show multiple local minima over the space of transformations, especially when working at low voxel sizes (Fig. 1), gradient based optimization techniques are not suitable.

#### Exhaustive search

Exhaustive search is a basic search algorithm where all candidates from the search space are sequentially generated and tested to find the solution which optimizes a certain criteria. To illustrate this with the example of estimating the rotational difference between two morphologies, exhaustive search can be formulated as sequentially generating all possible rotations, applying them to one of the morphologies, calculating spatial dissimilarity for each of them with the reference and choosing that rotation which leads to the least dissimilarity. However, the number of possible rotations is infinite. Therefore, an approximate estimate is obtained by generating a discrete set of equally spaced rotations from a plausible region of the rotation space and exhaustively searching among these rotations for the optimal rotation. This can be implemented by parametrizing rotation, sampling the plausible range of each parameter uniformly with a certain inter-sample-interval, and exhaustively searching all combinations of the resulting parameters (for implementation details see Additional file 2).

#### Multi-scale estimation

Using exhaustive search on a discretized search space imposes a trade-off between accuracy of the resulting estimate and the computational cost associated with its calculation. To reduce this computational cost, our algorithms use the strategy of hierarchical or multi-resolution matching [41, 42] which has been successfully used to speed up and reduce errors of 3D image registration methods. Starting at the largest voxel size, it runs an exhaustive search over an equally spaced discrete set of plausible parameters to find an estimate. The exhaustive search at the next lower voxel size is run over a smaller region around this estimate determined by its uncertainty (see Additional file 2 for more details). Thus estimates are progressively refined by running exhaustive searches over a sequence of discretized volumes generated using decreasing values of voxel size.

### Reg-MaxS

Using this multi-scale estimation method to determine transformation differences between morphologies, Reg-MaxS iteratively applies transformations to remove determined differences until no transformation reduces the spatial dissimilarity between the morphologies any further. It first translates one of the morphologies so that its center coincides with the other. It then applies a sequence of translation, rotation and scaling transforms to minimize the spatial dissimilarity between morphologies. The order in which the different transformations are applied is determined based on how the application of one transformation influences the subsequent estimation of another transformation difference.

Rotation and translation do not affect each other, i.e., if there are only rotation and translation differences between morphologies, it does not matter whether the rotation difference is removed first and then the translation difference or vice versa. However, scaling and rotation/translation affect each other, i.e., applying a scaling affects a subsequent estimation of a translation/rotation difference and vice versa. Hence, Reg-MaxS applies a sequence of translation/rotation transforms until no translation or rotation can reduce spatial dissimilarity further. Then it applies a scaling transform. This is followed again by a set of translation/rotation transforms which is then followed again by a scaling. This iteration of alternatively applying a set of translation/rotation and scaling is continued until none of the transforms can decrease the spatial dissimilarity between the morphologies any further. Finally, the iteration at which spatial dissimilarity was minimized is chosen as the final solution. (see Additional file 2 for actual algorithm). Note that Reg-MaxS does not handle reflections. Any reflections must be removed before the algorithm is applied.

### Reg-MaxS-N

Reg-MaxS-N is an algorithm for co-registering multiple morphologies. It uses Reg-MaxS for co-registering pairs of morphologies and is based on “iterative averaging” [43] which has been successfully used to generate several standard brain atlases [43–45]. It is an iterative algorithm, which in each iteration uses a reference volume and registers all morphologies to it. From the resulting registered morphologies, it generates an “average volume”, which is then used as the reference in the following iteration. For the first iteration, volume occupied of one of the morphologies to be registered is chosen as the initial reference. The iteration stops when all pairwise registrations of an iteration are rejected (see “Accepting a pairwise registration” section) Finally, the iteration at which the occupancy based measure of the morphologies was minimized is chosen as the final solution (see Additional file 2 for actual algorithm).

#### Computing the average volume

There are several ways of generating an average volume from a group of registered morphologies. In image stack registration paradigms, where voxel values are multi-valued and numerical (E.g.: for grayscale image stacks), an average of a set of images is generated by averaging the value for each voxel across the set of images. In other problems where voxel values are non-numerical (string labels for example, as in [43]), a democratic policy is used, where the most frequently occurring value is chosen for each voxel. However, in our formulation each voxel takes one of two values, ’1’ or ’0’, indicating whether it contains at least one point of the morphology or not. Using a democratic policy would mean that the average retains only those voxels for which more morphologies have ’1’s than ’0’s. For those cases where some parts of the morphologies have not yet overlapped at the end of the first iteration, this policy would remove those parts from the average. Since the morphologies are registered to this average in the following iteration, those parts would no longer be taken into account for registration. Instead, we use a more conservative approach and assign a voxel in the average volume to be ’1’ if at least one of the morphologies being averaged has a value ’1’. In other words, the average volume of a given set of morphologies is calculated as the union of the voxel sets of all the morphologies. This ensures that each morphology is completely represented in the average and thereby contributes equally in determining the final registration.

#### Initial approximate registration

For the first iteration, an initial approximate registration is performed by matching centroids. For all subsequent iterations, no initial registration is applied.

#### Restricting total scaling

In every iteration, Reg-MaxS-N uses Reg-MaxS for registering morphologies to an average volume. A parameter of Reg-MaxS is the range of values of scales over which Reg-MaxS searches to find the scale that, when applied to the test morphology, minimizes its spatial dissimilarity with the reference. However, if this range of possible scales is constant, and Reg-MaxS-N repeatedly aligns the morphologies to the average volume of the previous iteration, it would scale the morphologies larger and larger to stretch the dimensions which show high spatial dissimilarity. If such scaling is not constrained, the morphologies would become disproportionately and unrealistically large to achieve a high similarity value. Hence, Reg-MaxS-N constrains the total scaling that is applied to a morphology. It keeps track of the total scaling that has been already applied to a morphology at the end of each iteration and reduces the amount of scaling that can be applied to it in the next iteration. This prevents the total scaling from becoming unrealistic.

#### Normalizing final morphologies

As explained above, since Reg-MaxS-N repeatedly registers morphologies to the average of the previous iteration, the final morphologies would have translation, rotation and scaling differences with the initial reference morphology, i.e., the reference morphology of the first iteration. For further analysis on these final registered morphologies, it is convenient to transform them such that they are comparable to the original reference morphology. Thus, Reg-MaxS-N calculates the sum total of all translation, rotation and scaling transforms applied to the original reference morphology over all iterations and applies the inverse of this total transformation to all the final registered morphologies. This makes all of them comparable with the original reference morphology.

#### Accepting a pairwise registration

At each step, Reg-MaxS uses the multi-scale method for determining transformation differences. In the multi-scale method, the final estimate is determined at the lowest voxel size of the algorithm. Thus, Reg-MaxS tries to minimize spatial dissimilarity between two morphologies at this lowest voxel size. Doing so could lead to an increase in spatial dissimilarity at a higher voxel size. This is acceptable, since we want an exact or a very large overlap between the volumes of the morphologies. However, when working iteratively with a group of morphologies, the reference corresponds to an actual morphology only for the first iteration. For all other iterations, it is a conservative “average” representing the union of the volumes of several morphologies, which does not represent any single morphology. Sacrificing spatial overlap at a higher voxel size for spatial overlap at a lower voxel size can cause over-fitting, in the sense that parts which do not necessarily correspond to each other would end up being randomly matched. Hence, a morphology registered to an average is accepted only if spatial dissimilarity at the highest voxel size has decreased. If the spatial dissimilarity at the highest voxel size has remained the same, then the spatial dissimilarity at the next highest voxel size is considered, and so on. When a registration is not accepted, the test morphology is itself designated as the registered morphology.

### Testing the methods

To validate Reg-MaxS and Reg-MaxS-N, we tested them on several groups of morphologies. We defined measures for quantifying performance and calculated them for each of the test cases. Comparing these measures, we identified the cases where the algorithms performed poorly and investigated the reason behind them. In this section, we describe the morphologies and performance measures used for testing the algorithms.

#### Morphologies used for testing

##### Synthetic Morphologies used to test Reg-MaxS

**Fig. 2:**
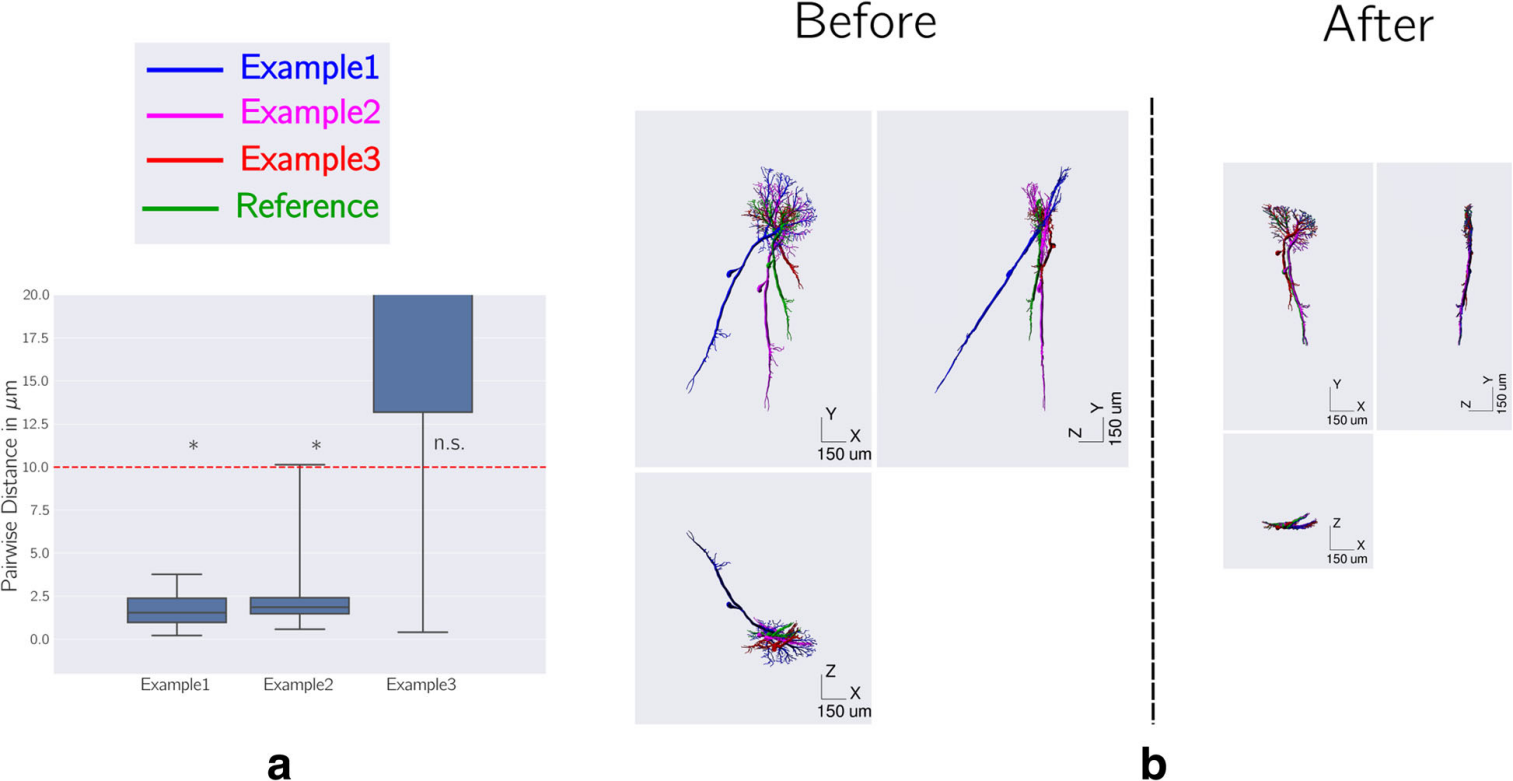
Examples of pairwise co-registration of morphologies using Reg-MaxS. Results of pairwise co-registration of a morphology (green) and three versions of it (blue, magenta and red) transformed by random translations, rotations and scaling. In each example, Reg-MaxS was applied to co-register a transformed morphology to the reference. **a** Distribution of corresponding point pairs distances between the resulting morphologies and the reference. Box plots extend between first and third quartiles with the median indicated by a black line while whiskers indicate the extrema. The red dashed line indicates the smallest voxel size used for the co-registrations. The Y-axis has been scaled to focus on distances in the range zero to the lowest voxel size, which indicate good registration performance. Asterisk indicates whether corresponding point pairs were significantly closer than the smallest voxel size used according to Signs test at 1% significance level. **b** The morphologies before and after co-registration. Reg-MaxS was successful in removing the transformation differences between the morphologies for Example1 and Example2 as shown by distribution of distances in (**a**) and close alignment in (**b**, “After”). For Example 3, which showed a high degree of anisotropic scaling (MAS=0.37), some scaling differences remained

To illustrate its working and explore its limitations, we applied Reg-MaxS to synthetic data generated from a morphology of a visual neuron from the blowfly [15] (Fig. 2b green) obtained from NeuroMorpho.org [2]. The morphology is nearly two dimensional and has a dense dendritic arbor with a thick axon which projects to a couple of nearby regions.

We first created a set of 10 noisy morphologies by adding independent zero-mean Gaussian noise of standard deviations (std) 1, 3, 5,...,17, 19 *μ*m to the points of the morphology. Next, 100 different random transformations were constructed by drawing translations from a uniform distribution over [-20, 20] *μ*m, rotations from a uniform distribution over [-30, 30] degrees and scaling from a uniform distribution over [0.5, 1/0.5]. Each transformation was applied to the set of ten noisy morphologies to generate one hundred such sets. In addition, 1000 noiseless morphologies were generated by applying 1000 different random transformations constructed as above to the original noiseless morphology. To summarize, we used 2000 transformed morphologies: (1000 without noise) + (100 with noise of std 1 *μm*) + (100 with noise of std 3 *μm*) +.... + (100 with noise of std 19 *μm*).

##### Morphologies used to test Reg-MaxS and Reg-MaxS-N

Table 1 describes the five groups of neuron morphologies from *Drosophila melanogaster* used for testing Reg-MaxS and Reg-MaxS-N. Morphologies within a group have stereotypic structure but each group shows a different three dimensional dendritic arborization (see Additional file 1).

**Table 1:** Neurons from *Drosophila melanogaster* used for testing Reg-MaxS and Reg-MaxS-N

Group name	No. of morphologies	Description	NBLAST Cluster [46]
LCInt	8	Interneuron of the fly Lobula complex	246
ALPN	14	Neuron projecting from the antennal lobe to the mushroom body	458
OPInt	23	Interneuron of the fly Optic lobe	209
AA1	12	Interneuron of fly ventrolateral protocerebum	921
AA2	9	Neuron of the fly antennal mechanosensory and motor center	803

All the morphologies were generated from image stacks of the FlyCircuit Database [31]. The morphologies reconstructed without registering to any standard brain atlas (“non-standard” morphologies) were obtained from NeuroMorpho.org [2]. Morphologies which were reconstructed after registering to a Drosophila standard brain [30, 46] (“standardized” morphologies) were obtained from Dr. Gregory Jefferis.

#### Measures for quantifying performance of Reg-MaxS

Reg-MaxS was evaluated by applying it to register a test morphology to a reference and calculating residual errors based on the Euclidean distances of corresponding point pairs between result and reference morphologies. When synthetic morphologies were used, the test morphologies were randomly transformed versions of the reference and hence a pointwise correspondence was readily available. When real morphologies were used, test and reference morphologies were from the group ‘LCInt’ and no such correspondence was available. In this case, correspondences were defined by choosing the nearest neighbor among the test SWC points for every SWC point of the reference morphology.

##### Measures of performance:

The residual error above between result and reference morphologies was quantified as follows. Given a reference morphology *P* and a result morphology *Q*_1_, let {*p*_1_,*p*_2_,⋯,*p*_*m*_} be the SWC points of *P* and $\{q_{p_{1}}, q_{p_{2}}, \cdots, q_{p_{m}}\}$ be their corresponding points in *Q*_1_. From these points, a set of Euclidean distances $\left \{d_{1}^{Q_{1}}, d_{2}^{Q_{1}}, \cdots, d_{m}^{Q_{1}}\right \}$ were calculated as follows:

$$\begin{array}{*{20}l} d_{i}^{Q_{1}} = & \sqrt{\left(p_{i}^{x} - q_{p_{i}}^{x}\right)^{2} + \left(p_{i}^{y} - q_{p_{i}}^{y}\right)^{2} + \left(p_{i}^{z} - q_{p_{i}}^{z}\right)^{2}} \\ & \text{for} \ i \ \text{in} \ \{1, 2, 3, \cdots, m\} \end{array} $$

where the superscripts *x*, *y* and *z* indicate coordinates in space. We used multiple tests for validation and therefore given a set of tests {*Q*_1_,*Q*_2_,*Q*_3_...,*Q*_*n*_}, a set of Euclidean distances as shown below were calculated.

$$\begin{aligned} \left\{ d_{1}^{Q_{1}}, \quad d_{2}^{Q_{1}}, \quad \cdots, \quad d_{m}^{Q_{1}},\right.\\[-2pt] d_{1}^{Q_{2}}, \quad d_{2}^{Q_{2}}, \quad \cdots, \quad d_{m}^{Q_{2}},\\[-2pt] \cdots, \quad \cdots, \quad \cdots, \quad \cdots,\\[-2pt] \left. d_{1}^{Q_{n}}, \quad d_{2}^{Q_{n}}, \quad \cdots, \quad d_{m}^{Q_{n}} \right\} \end{aligned} $$

Since the finest spatial scale at which Reg-MaxS registers morphologies is the smallest voxel size used, distances less than the smallest voxel size indicate good registration. We regrouped these distances in two ways to quantify two kinds of performances:

1. Performance for every test across SWC points, using
$\left \{\left \{d_{1}^{Q_{1}}, d_{2}^{Q_{1}}, \cdots, d_{m}^{Q_{1}}\right \}, \left \{d_{1}^{Q_{2}}, d_{2}^{Q_{2}}, \cdots, d_{m}^{Q_{2}}\right \}, \cdots, \left \{d_{1}^{Q_{n}}, d_{2}^{Q_{n}}, \cdots, d_{m}^{Q_{n}}\right \}\right \}$
2. Performance for every SWC point of the reference morphology across tests, using,
$\left \{\left \{d_{1}^{Q_{1}}, d_{1}^{Q_{2}}, \cdots, d_{1}^{Q_{n}}\right \},\left \{d_{2}^{Q_{1}}, d_{2}^{Q_{2}}, \cdots, d_{2}^{Q_{n}}\right \}, \cdots, \left \{d_{m}^{Q_{1}}, d_{m}^{Q_{2}}, \cdots, d_{m}^{Q_{n}}\right \}\right \}$

These performance measures were calculated as the percentage of tests or SWC points for which distances were significantly smaller than the smallest voxel size used. Since only distance values smaller than the smallest voxel size were relevant, we used the one-tailed Wilcoxon test, also known as the Signs test with a significance level cutoff of one percent.

##### Measure of anisotropic scaling:

Some preliminary tests with Reg-MaxS indicated that performance of the algorithm was affected by different scaling along different axes of the morphologies relative to each other (see “Results” section). To quantify such differences in scaling along axes, we defined the following Measure of Anisotropic Scaling (MAS):

$$ \text{MAS} = 1 - \frac{1}{3}\left(\frac{s_{1}}{s_{2}}+\frac{s_{1}}{s_{3}}+\frac{s_{2}}{s_{3}}\right) $$

where *s*_1_,*s*_2_,*s*_3_ are the scaling differences along the axes arranged in ascending order. MAS has a value of zero when the scaling differences along all axes are equal, and increase gradually to one as the scales become more and more different.

### Comparing Reg-MaxS-N with other methods

We compared the performance of Reg-MaxS-N with Reg-MaxS and four other methods for co-registering morphologies from recent studies:

- **PCA:** A method using Principal Component Analysis based on a similar method for image stacks [47].
- **PCA + RobartsICP:** The PCA method above followed by Anisotropic-Scaled Iterative Closed Point [36].
- **BlastNeuron:** The affine transformation step of BlastNeuron [33].
- **Standardized:** A method using a standard brain [30].

Code for BlastNeuron and RobartsICP was obtained from the respective authors. Morphologies registered to a standard brain were provided by Dr. Gregory Jefferis. The PCA method was implemented as follows. Given a test and a reference morphologies, we assumed that they have similar dendritic density profiles and were oriented similarly in space. Based on this, the method assumes a correspondence between the first principal axes (principal axes corresponding to the largest principal factors), second principal axes and the third principal axes of the two morphologies. This method translates the test morphology so that its center coincides with that of the reference and rotates it so that their corresponding principal axes align. Scaling differences are determined based on the variances of the morphologies along the corresponding principal axes and the test morphology is appropriately scaled.

Each registration method was applied to each of the five groups of morphologies with the standardized version of one of the morphologies as the initial reference. Performance was quantified using the occupancy-based measure defined above. The results of PCA, PCA + RobartsICP, Reg-MaxS and Reg-MaxS-N were in the same frame of reference as the standardized morphologies allowing direct comparison. The results of BlastNeuron however were in a different frame of reference.

In addition, the above registration tests were repeated three times for each method and each group using different morphologies as initial references and performances were quantified in each case.

### Computing density profiles from sets of registered morphologies for visualization

We visualized the results of PCA, BlastNeuron and Reg-MaxS-N along with the standardized morphologies by constructing density profiles from each of them and by maximal projections of these density profiles along two orthogonal planes. These density profiles were generated using the method described in [30]. For each set of morphologies that were co-registered, a density profile was constructed discretized with a voxel size of 0.25*μm*×0.25*μm*×0.25*μm*. Each morphology was resampled so that the distance between any pair of connected points was at most 0.1*μm*. Each voxel that contained at least one point of the morphology was assigned a value of 1 and all others were assigned 0. This binary density profile was smoothed using a unity sum 3D discrete Gaussian Kernel. The standard deviation of this kernel was chosen individually for each group of morphologies. Density profiles so calculated for each morphology were averaged across morphologies to obtain a density profile for the set of morphologies.

## Results

### Testing Reg-MaxS with synthetic morphologies

#### Testing Reg-MaxS with noiseless morphologies

We first used the synthetically generated noiseless morphologies for testing Reg-MaxS. In each of these test registrations, the respective original morphology was always used as the reference while a transformed version of the original morphology was used as the test. The smallest voxel size used was 10 *μm* for all the tests. When pointwise distance statistics were calculated for each test registration across SWC points, 675 of 1000 tests (67.5%) had final distances that were significantly smaller than the smallest voxel size (n =1290, Signs Test, 1% significance level). When pointwise distance statistics were calculated for each SWC point across test registrations, 1287 of 1290 SWC points (99.76%) had final distances that were significantly smaller than the smallest voxel size (n =1000, Signs Test, 1% significance level). Thus, although Reg-MaxS fails to register a significant number of SWC points in a third of the test registrations, the number of points for which it consistently fails across tests is small.

Three example tests are illustrated in Fig. 2. Reg-MaxS failed for the test morphology “Example3”, especially in removing scaling differences. This was caused by the heavy anisotropic scaling in this morphology (scaling differences: 1.12 along X, 0.61 along Y and 1.27 along Z, MAS =0.37). We analyzed this further by separating morphologies based on their level of anisotropic scaling (see “Effect of anisotropic scaling” section below).

In these tests the morphologies used had nearly planar densities. However, Reg-MaxS also performed well on morphologies with 3D extent. This is demonstrated in the “Testing Reg-MaxS with real reconstructions” section using LCInt morphologies which have a non-planar dendritic density profile.

#### Effect of anisotropic scaling

To investigate the effect of the level of anisotropic scaling on the performance of Reg-MaxS, we calculated statistics only for the tests with low levels of anisotropic scaling, i.e., for cases where Measure of Anisotropic Scaling (MAS) was less than 0.2. Across SWC points, 166 of 193 tests (86%) had significant numbers of final distances smaller than the smallest voxel size (n =1290, Signs Test, 1% significance level). Across test registrations, 1290 of 1290 SWC points (100%) had final distances less than smallest voxel size (n =193, Signs Tests, 1% significance level). This shows that Reg-MaxS performs better for cases with low levels of anisotropic scaling, i.e, for cases where the MAS is less than 0.2.

#### Testing Reg-MaxS with noisy morphologies

Reg-MaxS was designed to co-register morphologies so that their spatial characteristics can be compared, assuming that the morphologies have very similar structure and belong to the same stereotypic neuron group but are obtained from different specimens. Even stereotypical neurons exhibit natural biological variability in the exact location of their dendrites from individual to individual, especially for higher order dendrites. Thus, in order to properly register such morphologies, Reg-MaxS must be able to tolerate such variability in dendritic position. We tested this by applying Reg-MaxS to morphologies where noise was added to each point of the morphology.

**Fig. 3:**
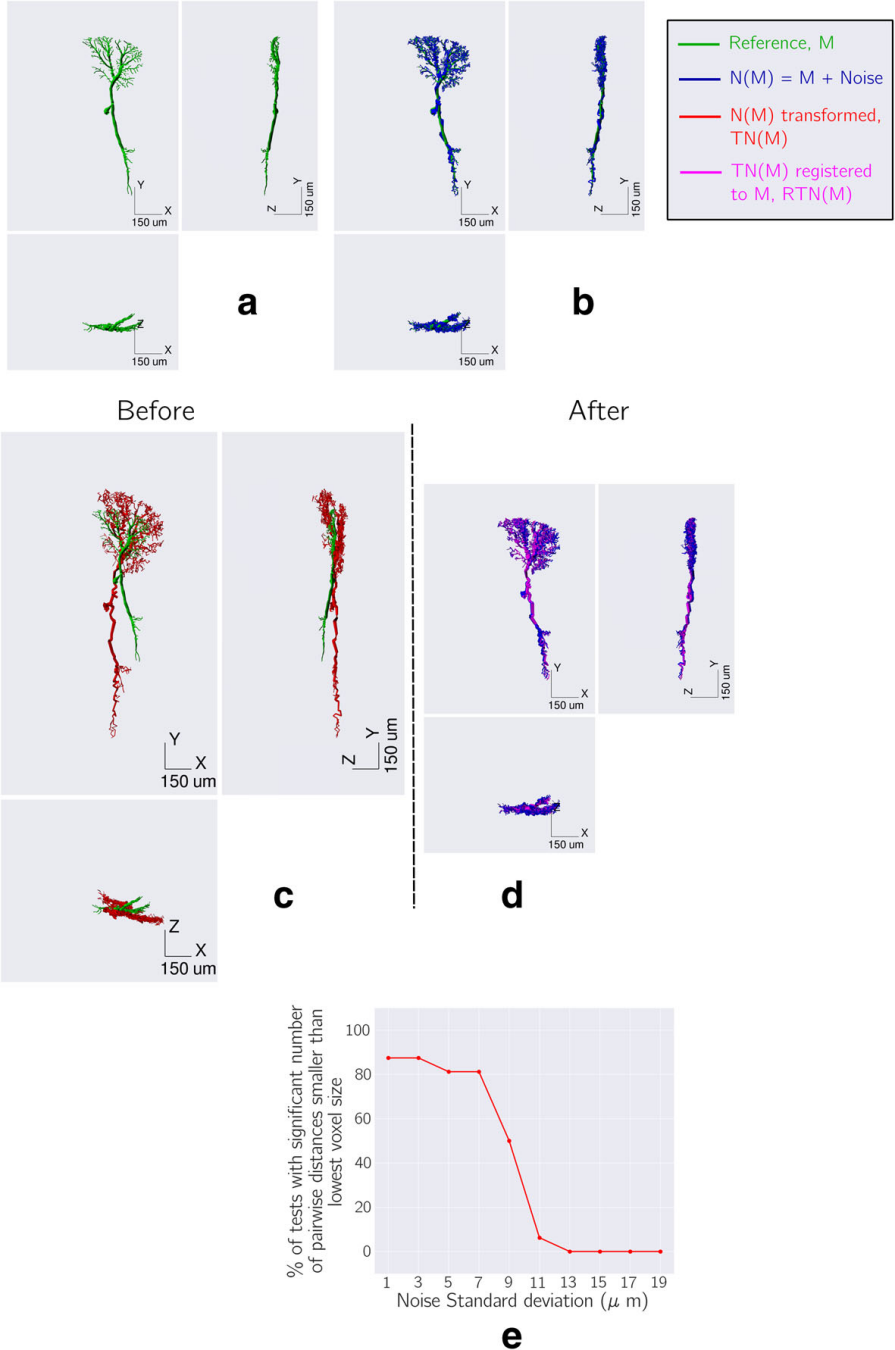
Testing Reg-MaxS with noisy morphologies. **a** The reference morphology M. **b** M (green) and the morphology N(M) (blue), which was obtained by adding independent Gaussian noise of standard deviation 7 *μm* to each point of M. **c** M (green) and the morphology TN(M) (red), which was obtained by applying random translation, scaling and rotation to N(M). **d** N(M) (blue) and RTN(M) (violet), which was obtained by registering TN(M) to M using Reg-MaxS. The process was repeated using multiple random transformations and different values of noise standard deviations (see “Methods” section). **e** Performance of Reg-MaxS as a function noise standard deviation. Reg-MaxS performance was calculated as the percentage of tests for which the distribution of resulting pointwise distances was significantly smaller than the smallest voxel size (10 *μm*). Reg-MaxS-N showed high performance for noise with standard deviation below the smallest voxel size

As described in “Methods” section, we generated noisy synthetic morphologies by first adding independent Gaussian noise to each point of a reference morphology M (Fig. 3a) to generate a noisy morphology N(M), shown in Fig. 3b. Then we randomly transformed N(M) to obtain the morphology TN(M), shown in Fig. 3c together with the original morphology M. We then ran Reg-MaxS with M as reference and TN(M) as the test to produce the morphology RTN(M), shown in Fig. 3d. Since the best expected registration of TN(M) to M is N(M), we compared RTN(M) to N(M) and calculated point-wise distances and registration accuracy accordingly. This was done for ten different values of standard deviation and a hundred different transforms. Figure 3e show the results of these tests. Reg-MaxS showed about 85% success for values of noise standard deviation less than the smallest voxel size.

### Testing Reg-MaxS with real reconstructions

Reg-MaxS applies affine transforms for reducing spatial dissimilarity between morphologies. However, multiple morphologies of the same stereotypical neuron obtained from different specimens could show non-affine differences as well, if the brains of the specimens show non-affine differences. This is taken into account while constructing brain atlases that use both affine and non-affine transforms (e.g., [43]). To test if the limitation to affine transforms is a major drawback for Reg-MaxS, we registered non-standard versions of LCInt morphologies (see Additional file 1 for its 3D structure) to their corresponding standardized versions. Since a pointwise correspondence between the morphologies was not available in this case, we used distance statistics of nearest point pairs of the reference morphology and the registered morphology for quantifying algorithm performance. The algorithm performed well on all neurons, with significant number of nearest point pairs closer than the smallest voxel size (117≤*n*≤276, Signs test, 1% significance level). However, these tests showed slightly larger final distances (5.51 ± 4.49 *μm*) compared to tests using noiseless synthetic morphologies with only affine transformation differences (3.08 ± 3.35 *μm*). The distributions of nearest point distances also showed more outliers compared to noiseless synthetic tests because of non-rigid differences between the non-standard and standardized morphologies.

### Testing Reg-MaxS-N with groups of morphologies

**Fig. 4:**
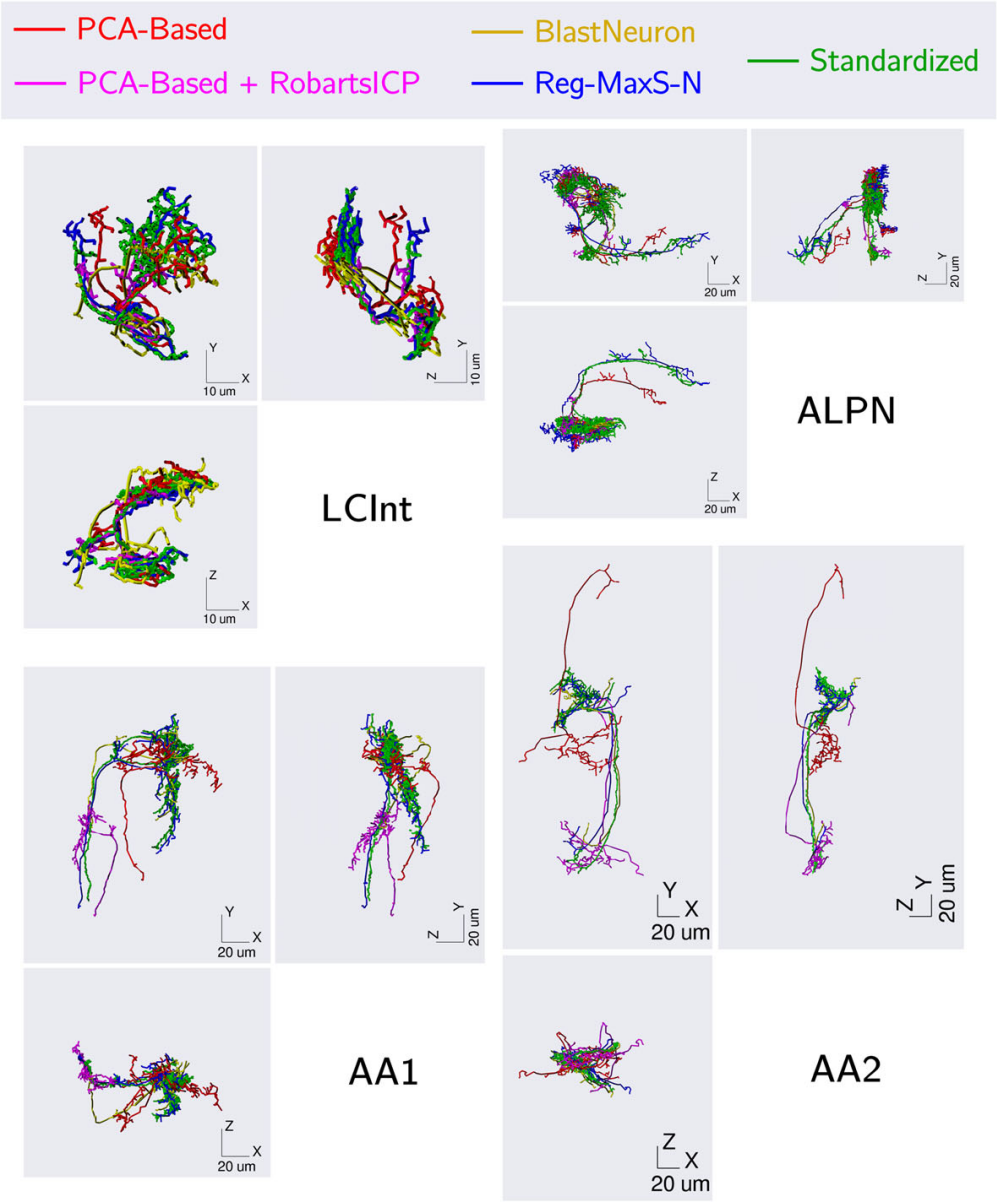
Comparative visualization of co-registration results of different methods using single morphologies. Co-registration results of PCA, PCA+RobartsICP, BlastNeuron, Reg-MaxS-N and standard brain based method visualized using a single morphology for LCInt (**a**), ALPN (**b**), AA1 (**c**) and AA2 (**d**). These visualizations illustrate some of the misalignments that can occur from the application of these methods

**Fig. 5:**
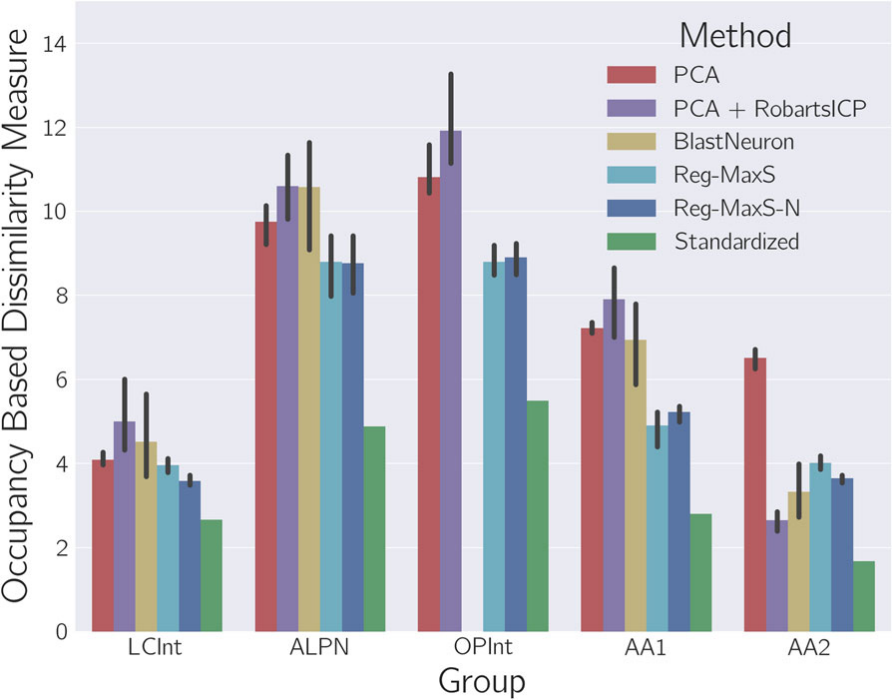
Performance comparison of registration methods. Performance of six registration methods for five different groups of morphologies. Error bars indicate 95% confidence intervals of median performance, calculated across values for four initial references. BlastNeuron performance for OPInt morphologies are not shown as the program provided by the authors stopped after a time limit of 30 min and produced no results. In most cases, Reg-MaxS and Reg-MaxS-N outperform the other methods

**Fig. 6:**
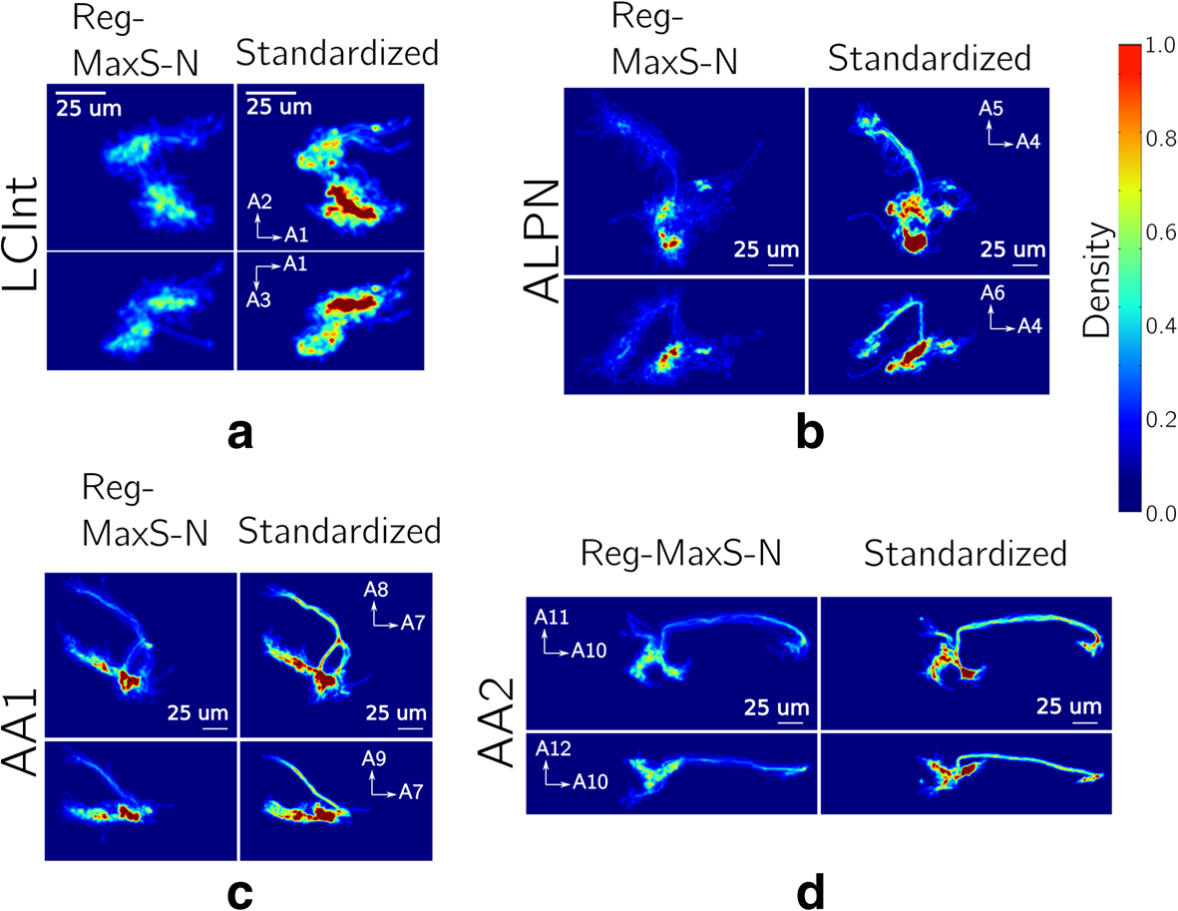
Comparison of dendritic density profiles generated using Reg-MaxS-N and brain altas based method. **a** Two dimensional projections of morphological densities after registration with Reg-MaxS-N (left columns) and standard brain based registration (right columns). Densities were calcuated for voxels of size 0.25*μm*. Color indicates the fraction of morphologies that, after registration, had at least one dendrite in the particular voxel. For illustration purposes, densities have been smoothed by a 3D Gaussian kernel with standard deviation of 1.25*μm*. **a** Densities for eight LCInt morphologies. A1, A2 and A3 correspond to the principal axes of the standardized LCInt morphology used as initial reference. **b** Densities for fourteen ALPN morphologies. A4, A5 and A6 correspond to the principal axes of the standardized ALPN morphology used as initial reference. **c** Densities for twelve AA1 morphologies. A7, A8 and A9 correspond to the principal axes of the standardized AA1 morphology used as initial reference. **d** Densities for nine AA2 morphologies. A10, A11 and A12 correspond to the principal axes of the standardized AA2 morphology used as initial reference. In all cases, Reg-MaxS-N produced densities very similar to that produced by standard brain based method

For evaluating Reg-MaxS-N, we compared its performance with that of five other methods (see “Methods” section). We applied the six methods to five groups of morphologies, repeating each case for four different initial references. Results of applying the methods are visualized in Fig. 4 using one sample morphology per group. Performance was quantified using occupancy-based dissimilarity (see “Methods” section) and averaged across initial references as shown in Fig. 5. Reg-MaxS-N outperformed PCA, BlastNeuron and PCA+RobartsICP for four of the five groups – LCInt, ALPN, OPInt and AA. For AA2, a group of neurons with unusually high structural stereotypy, BlastNeuron and PCA+RobartsICP showed slightly higher performance than Reg-MaxS-N (see “Applicability” in “Discussion” section for more). The density profiles calculated from the result morphologies of Reg-MaxS-N were very similar to those obtained using methods relying on a standard brain (Fig. 6). Furthermore, the performance of Reg-MaxS-N across initial references was less variable than BlastNeuron, PCA-Based+RobartsICP and Reg-MaxS for all groups as seen from the error bars in Fig. 5 (also see Additional file 3). Although PCA showed lower variance across initial references for ALPN and AA1 morphologies, its median performance was lower. Thus Reg-MaxS-N showed higher average performance and lower sensitivity to initial reference than other existing methods in a large majority of our tests.

## Discussion

We have presented Reg-MaxS and Reg-MaxS-N, algorithms for co-registering pairs and groups of neuron morphologies, respectively, by maximizing spatial overlap. We have quantified the performance of Reg-MaxS using synthetic and real morphologies. We have tested Reg-MaxS-N on different groups of morphologies with different initial references and quantified its performance for each case.

### Initialization

Spatial registration is a global optimization problem usually consisting of multiple local minima. Most registration algorithms therefore initialize using an approximate solution before minimizing dissimilarity. Several different strategies have been developed for initialization of registration algorithms [48]. However, initialization is required only when the objects being registered are expected to have large transformation differences. Neuron morphologies of the same type obtained from different individuals do no usually have large transformation differences other than translations caused by arbitrary choice of origin. Hence Reg-MaxS uses centroid alignment for initialization. Nonetheless, Reg-MaxS can be easily modified to include an appropriate initialization if an application demands it.

### Reg-MaxS vs Reg-MaxS-N

Compared to Reg-MaxS, Reg-MaxS-N has mainly two additional components in its procedure — iterative registration and final normalization. While Reg-MaxS registers all morphologies once to the initial reference, Reg-MaxS-N applies multiple iterations of such registrations, calculating a new reference in each iteration. This iterative strategy reduces the effect of the choice of initial reference on algorithm performance. In our tests, Reg-MaxS-N performed better than Reg-MaxS for most cases, and showed less variability across different initial references compared to Reg-MaxS (see Figs. A31 and A35 of Additional file 3), indicating better suitability for these cases.

For ALPN, OPInt and AA1 morphologies, the performance of Reg-MaxS-N was nearly the same as that of Reg-MaxS. In these cases, Reg-MaxS-N chose the morphologies at the end of its first iteration as the solution, i.e., the same solution as Reg-MaxS. However, the solution morphologies for Reg-MaxS-N were additionally normalized so that they were comparable to the initial reference and this caused the observed reduction in performance of Reg-MaxS-N compared to Reg-MaxS in some of these cases. The normalization was applied mainly for the purpose of visualization and comparison with other methods, and can therefore be excluded when analyzing single groups of morphologies.

### Computational cost

Reg-MaxS applies a sequence of transformations for maximizing spatial overlap between two morphologies. It estimates transformation differences at each step using a measure of spatial overlap based on the set of voxels occupied by each morphology. However, the set of voxels occupied by a morphology can change with every rotation or scaling. This makes it hard to predict the computational cost of estimating transformation differences at each step and thus to estimate the total computational cost of Reg-MaxS. Furthermore, Reg-MaxS and Reg-MaxS-N are both iterative algorithms which stop only when spatial overlap between morphologies cannot be improved further. This further complicates the prediction of total number of iterations and total computational cost.

We compared the run times per morphology of Reg-MaxS, Reg-MaxS-N and other methods for co-registration of groups of neurons with different settings of initial reference (Table 2). Run times for Reg-MaxS-N were many times longer than those of the other methods. This is because Reg-MaxS-N iteratively registers morphologies many times, refining their spatial alignment and incorporating features of all morphologies. Therefore Reg-MaxS-N is expected to run longer than methods that register each morphology to the reference only once. A more suitable comparison is between Reg-MaxS and the other methods, since all of them register each morphology once. These run times were comparable, differing by factors between 0.25 and 5. The main reason for the variabilities seen both in the run times of each algorithm and in the relative run times between algorithms is that all the algorithms except PCA use iterative routines for finding optimal transformations and their run times can vary substantially and differently depending on properties of the morphologies like size, number of SWC nodes and spatial structure.

**Table 2:** Comparison of average runtimes per morphology for different registration algorithms

	Average runtimes per morphology (s)				
Method	LCInt	ALPN	OPInt	AA1	AA2
PCA	0.07	0.08	0.45	0.59	0.25
PCA + RobartsICP	189.63	318.18	287.02	68.07	39.03
BlastNeuron	19.48	319.32	N.A.	115.95	185.78
Reg-MaxS	98.44	239.20	474.88	90.45	46.89
Reg-MaxS-N	1141.81	605.82	2796.96	883.670	633.44

The runtimes per morphology for each method and test group averaged across initial references. The runtimes of BlastNeuron for OPInt group of morphologies were unavailable as the program provided by the authors of BlastNeuron stopped execution after an internally defined time limit without producing output

### Choice of voxel sizes

The most important parameters of Reg-MaxS and Reg-MaxS-N are the set of voxel sizes over which transformation difference estimates are refined during co-registration of morphologies. The largest and the smallest voxel sizes define the coarsest and the finest spatial scales, respectively, at which the algorithms register morphologies. The algorithms consider a voxel to be occupied by a morphology if it contains one or more of its SWC points and align morphologies by applying transformations to match the sets of occupied voxels. Thus morphological features at scales finer than that defined by the smallest voxel size are ignored by the algorithms. Therefore, a good choice for the smallest voxel size is the spatial scale below which morphological features are not expected to match.

In our preliminary tests involving morphologies of different sizes and dendritic densities, we found a smallest voxel size of 10*μm* to be a good compromise and therefore used it for evaluating algorithm performances. To investigate the effect of reducing the value of the smallest voxel size, we repeated the tests by setting the value of smallest voxel size to 5*μm*. The results are summarized in Additional file 4. For pairwise co-registration of test morphologies that were larger in size and that had fewer features at scales smaller than 10*μm* than other test morphologies, the performance of Reg-MaxS reduced from 67.5% at 10*μm* to 32.2% at 5*μm*. On the other hand, for pairwise co-registration of test morphologies that were smaller in size and had more features at scales smaller than 10*μm*, performance of Reg-MaxS showed only a minor improvement. Furthermore, performance of Reg-MaxS and Reg-MaxS-N in co-registration of groups of morphologies did not show any substantial changes when smallest voxel size was changed from 10*μm* to 5*μm* (Additional file 4, Fig. A41). Thus, the value of smallest voxel size can influence the performance of our algorithms depending on the size and the sparsity of structural features of morphologies being registered, and should be chosen accordingly.

### Applicability

Reg-MaxS repeatedly applies a set of rotation/translations followed by a scaling to maximize spatial overlap between morphologies. Scales are estimated after aligning centroids of morphologies. In other words, Reg-MaxS seeks a solution of close centroid alignment. Therefore Reg-Max-S and consequently Reg-MaxS-N are best applicable to morphologies that are complete and have similarly situated centroids. Their application to partial morphologies or largely incomplete reconstructions is not straightforward and requires caution and consideration. For more efficient handling of such cases, the algorithms could be modified so that they do not depend heavily on centroid alignment.

Reg-MaxS-N was outperformed by PCA + RobartsICP and BlastNeuron for one out of five of our test groups of morphologies, AA2. Importantly, this was not due to poor performance of Reg-MaxS-N, but due to untypically good performance of BlastNeuron and PCA+RobartsICP. A reason for this could lie in the unusually high structural stereotypy of AA2 morphologies, which is also reflected by lower values of occupancy-based dissimilarity compared to other groups (Fig. 5, also see Fig. A35 of Additional file 3). This high structural stereotypy indicates the existence of a solution with very close point-to-point alignment, and hence BlastNeuron and PCA + RobartsICP, which are based on pointwise distance statistics, performed better. Under most realistic conditions, however, neuron morphologies will have a non-negligible biological variability in their fine spatial features, and therefore we would expect Reg-MaxS-N to perform better than the other methods considered here, as was the case for the other four test groups. However, since our sample sizes were small (n =4) we could not establish statistical significance for the differences in performance.

### Calculating dendritic density profiles using Reg-MaxS-N

Applying Reg-MaxS-N to three groups of stereotypic neuron morphologies from the *Droshophila melanogaster* brain, we have shown that Reg-MaxS-N can co-register groups of neuron morphologies. Without the need for an external reference like a standard brain atlas, the registration results were very similar to morphologies registered conventionally, using such a reference. Dendritic density profiles can be calculated from groups of registered morphologies by spatial averaging (see “Methods” section). Thus Reg-MaxS-N can be used to calculate dendritic density from profiles of stereotypic neurons (Fig. 6). Such density profiles are useful in analyzing spatial variances in different subregions of neurons and can provide insights about the brain regions surrounding neurons [11]. Furthermore, density profiles so calculated could be used in generative models of neuron morphology [10, 49, 50]. Such models usually assume simple density profiles like a uniform density over the region of arborization. The availability of better spatial density profile estimates can improve such existing models and also enable the development of new models.

### Possible improvements

Reg-MaxS applies a sequence of translation, rotation and scaling transformations to maximize the spatial overlap between morphologies. We tested Reg-MaxS with synthetic morphologies that had random translation, rotation and scaling differences and demonstrated its ability to revert these transformations. Other affine differences like shear would be expected to be compensated approximately by combinations of rotation and anisotropic scaling transformations. However, specifically including shear in the sequence of transformations applied could speed up the registration process and possibly result in better performance.

Topological features play an important role in determining neuronal function [51, 52] and hence are indispensable in the study of neuron morphology. Some recent studies [26, 33] have illustrated the effectiveness of the combined use of spatial and topological features for characterization and classification of morphology. Since Reg-MaxS-N can provide better spatial registration of morphologies than existing methods, it could be used as preprocessing to remove spatial differences for algorithms that subsequently estimate topological differences. Further, incorporating topological features into its formulation could lead to even more powerful methods for analyzing neuron morphologies.

## Conclusion

We have addressed the problem of co-registering neuron morphologies, which is a crucial requirement for visualization and spatial analysis of stereotypical neurons, by formulating algorithms based on maximizing spatial overlap. Our tests using synthetic and real groups of morphologies have indicated that our algorithms can be used for registering stereotypic neuron morphologies that show considerable spatial variability in their fine structures as long as they are similarly scaled along different axes. The dendritic densities of stereotypic neurons calculated using our algorithms were very similar to those produced using a standard brain, demonstrating the potential of our algorithms in detailed spatial comparison of neuron morphologies.

## Additional files

Additional file 1 Figure showing the five groups of neuron morphologies used for evaluating Reg-MaxS-N registered to a standard brain (a) Interneurons in the Lobula complex (b) Antennal lobe projection neurons (c) Interneurons of ventrolateral protocerebum (d) Neuron of the antennal mechanosensory and motor center (e) Interneurons in the optic lobe. Figures from http://flybrain.mrc-lmb.cam.ac.uk/si/nblast/clusters/. (PNG 2101 kb)

Additional file 2 Implementation details of Reg-MaxS and Reg-MaxS-N. (PDF 306 kb)

Additional file 3 Performance comparison of Reg-MaxS-N and other methods for different initial references plotted separately for each group of morphologies. (PDF 593 kb)

Additional file 4 Tests Results of Reg-MaxS and Reg-MaxS-N with smallest voxel size set to 5*μm*. (PDF 181 kb)
