# Supplementary figures and images for: Spatial registration of neuron morphologies based on maximization of volume overlap
Source: BMC Bioinformatics. 2018 Apr 18;19:143. doi: 10.1186/s12859-018-2136-z (PMC5907365; doi:10.1186/s12859-018-2136-z)

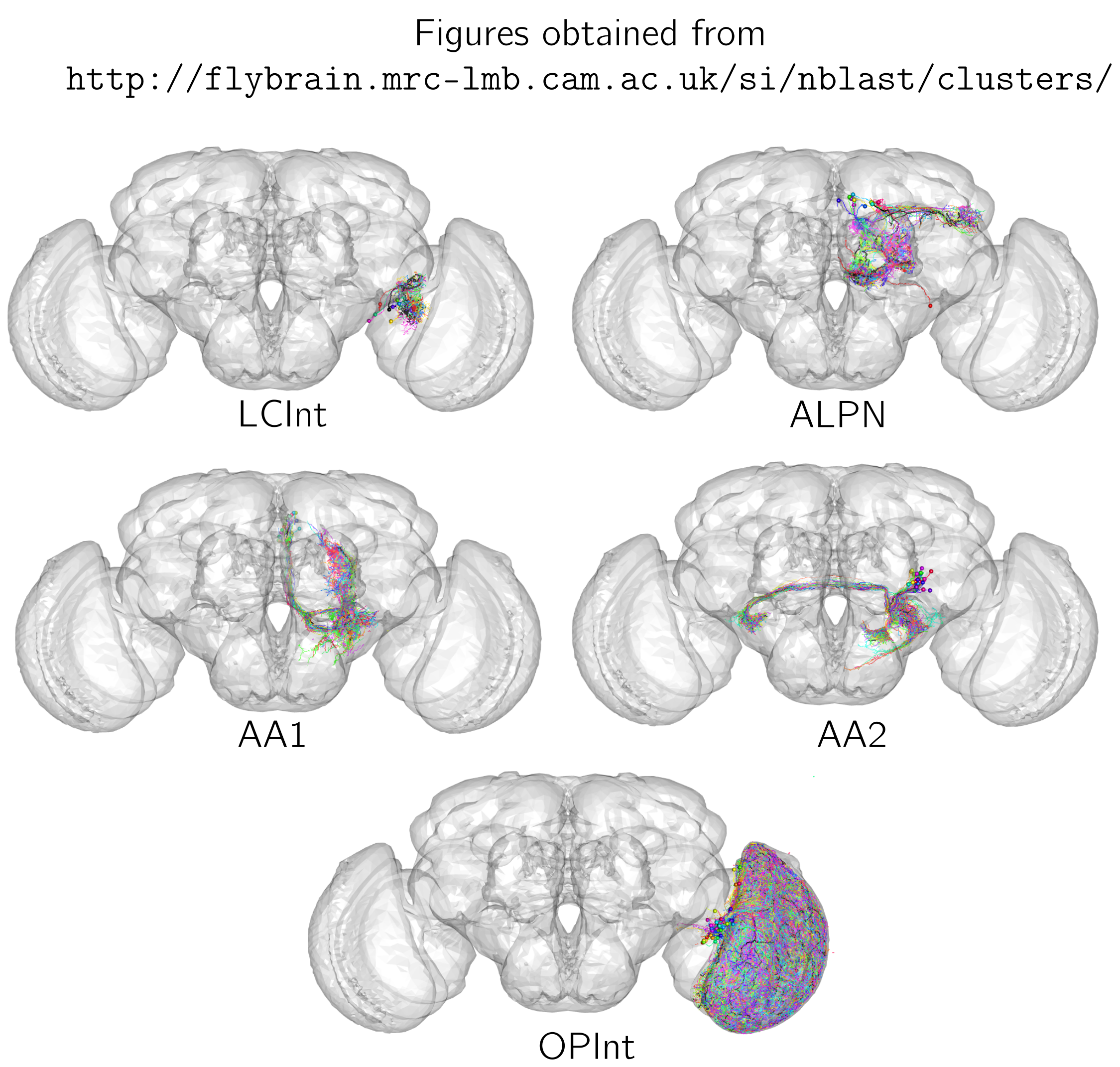

Supplement: Supplementary file 1 — Figure showing the five groups of neuron morphologies used for evaluating Reg-MaxS-N registered to a standard brain (a) Interneurons in the Lobula complex (b) Antennal lobe projection neurons (c) Interneurons of ventrolateral protocerebum (d) Neuron of the antennal mechanosensory and motor center (e) Interneurons in the optic lobe. Figures from http://flybrain.mrc-lmb.cam.ac.uk/si/nblast/clusters/. (PNG 2101 kb) [file 12859_2018_2136_MOESM1_ESM.png]
