# Supplementary material for: Spatial registration of neuron morphologies based on maximization of volume overlap
Source: BMC Bioinformatics. 2018 Apr 18;19:143. doi: 10.1186/s12859-018-2136-z (PMC5907365; doi:10.1186/s12859-018-2136-z)
Supplement: Supplementary file 2 — Implementation details of Reg-MaxS and Reg-MaxS-N. (PDF 306 kb) [file 12859_2018_2136_MOESM2_ESM.pdf]

Additional File 2: Implementation Details of Reg-MaxS and Reg-MaxS-N

as part of "Spatial registration of neuron morphologies based on maximization of volume overlap"

Ajayrama Kumaraswamy*[1], Kazuki Kai[2], Hiroyuki Ai[2], Hidetoshi Ikeno[3], and Thomas Wachtler†[1]

[1]Dept. Biologie II, Ludwig-Maximilians-Universität München
[2]Department of Earth System Science, Fukuoka University, Fukuoka, Japan
[3]School of Human Science and Environment, University of Hyogo, Himeji, Japan

Transformations

To minimize the spatial dissimilarity of one morphology with another, we apply three kinds of transformations, namely, translation, rotation and scaling. We define these transformations here, as they apply to voxel sets.

Let $\{\mathbf{a}_\mathrm{i}\}$ be the set of voxels corresponding to the morphology being transformed. Each voxel $\mathbf{a}_\mathrm{i}$ of set $\{\mathbf{a}_\mathrm{i}\}$ is a three dimensional vector represented as $[ax_i, ay_i, az_i]^T$ containing the X, Y and Z coordinates of the centroid of the voxel respectively. The mean of all the points of set $\{\mathbf{a}_\mathrm{i}\}$ is $\mathbf{a}_\mathrm{m}$.

A translational transformation is represented by three parameters dx, dy and dz which are added to the X, Y and Z coordinates, respectively, of each point of the set being transformed. For each point $\mathbf{a}_\mathrm{i}$, the translated point $\mathbf{t}_\mathrm{i}$ is given by:

$$\mathbf{t}_\mathrm{i} = \mathbf{a}_\mathrm{i} + [dx, dy, dz]^T$$

A rotational transformation is represented by three parameters θ_x, θ_y and θ_z which are the rotations applied about X, Y and Z axes respectively. Each of these rotations are applied about the centroid of the corresponding morphology and the order of application is XYZ. For each point $\mathbf{a}_\mathrm{i}$, the rotated point $\mathbf{r}_\mathrm{i}$ is defined as:

$$\begin{aligned} R &= f_R(\theta_x, \theta_y, \theta_z) \\ \mathbf{r}_\mathrm{i} &= R\left(\mathbf{a}_\mathrm{i} - \mathbf{a}_\mathrm{m}\right) + \mathbf{a}_\mathrm{m} \end{aligned}$$

where f_R is the function which generates the rotation matrix R from the rotation parameters θ_x, θ_y and θ_z.

A scaling transformation is represented by three values s_x, s_y and s_z which are the scaling applied along the X, Y and Z axes, respectively. These scales are applied about the centroid of the corresponding morphology. For each point $\mathbf{a}_\mathrm{i}$, the scaled point $\mathbf{s}_\mathrm{i}$ is defined as:

$$\begin{aligned} S &= f_S(s_x, s_y, s_z) \\ \mathbf{s}_\mathrm{i} &= S\left(\mathbf{a}_\mathrm{i} - \mathbf{a}_\mathrm{m}\right) + \mathbf{a}_\mathrm{m} \end{aligned}$$

where f_S is the function which generates the scaling matrix S from the scaling parameters s_x, s_y and s_z.

*ajayramak@bio.lmu.de
†wachtler@bio.lmu.de

Choosing a discrete set of parameters for exhaustive search

The set of discrete values for a transformation parameter is chosen centering on an existing estimate, when one is available, or about [0, 0, 0] (for translation and rotation) or [1, 1, 1](for scaling) when there is none. This is done for each parameter axis separately, by successively adding or subtracting a certain step size from the center for translation and rotation, and by successively dividing and multiplying the center with a step size for scaling until the bounds are reached. This is illustrated in the following sections.

Translation and Rotation:

Given a previous estimate $w = [t_x, t_y, t_z]$, a step size Δ and upper and lower bounds $[l, u]$ for each parameter, the translation parameter axes are sampled as follows:

$$\begin{aligned}
\text{Along } X: &\quad [lf_x, lf_x + \Delta, lf_x + 2\Delta, \cdots, t_x - \Delta, t_x, t_x + \Delta, \cdots, uc_x - 2\Delta, uc_x - \Delta, uc_x] \\
\text{Along } Y: &\quad [lf_y, lf_y + \Delta, lf_y + 2\Delta, \cdots, t_y - \Delta, t_y, t_y + \Delta, \cdots, uc_y - 2\Delta, uc_y - \Delta, uc_y] \\
\text{Along } Z: &\quad [lf_z, lf_z + \Delta, lf_z + 2\Delta, \cdots, t_z - \Delta, t_z, t_z + \Delta, \cdots, uc_z - 2\Delta, uc_z - \Delta, uc_z]
\end{aligned}$$

where $lf_x = t_x + \Delta * floor((l - t_x)/\Delta)$, similarly for lf_y and lf_z; and $uc_x = t_x + \Delta * ceil((u - t_x)/\Delta)$, similarly for uc_y and uc_z.

Scaling:

Given a previous estimate $w = [s_x, s_y, s_z]$, a step size Δ (see below), and bounds $[l, u]$ for each parameter, the scaling parameter axes are sampled as follows:

$$\begin{aligned}
\text{Along } X: &\quad [lf_x, lf_x * \Delta, lf_x * \Delta^2, \cdots, t_x/\Delta, t_x, t_x * \Delta, \cdots, uc_x/\Delta^2, uc_x/\Delta, uc_x] \\
\text{Along } Y: &\quad [lf_y, lf_y * \Delta, lf_y * \Delta^2, \cdots, t_y/\Delta, t_y, t_y * \Delta, \cdots, uc_y/\Delta^2, uc_y/\Delta, uc_y] \\
\text{Along } Z: &\quad [lf_z, lf_z * \Delta, lf_z * \Delta^2, \cdots, t_z/\Delta, t_z, t_z * \Delta, \cdots, uc_z/\Delta^2, uc_z/\Delta, uc_z]
\end{aligned}$$

where $lf_x = t_x * \Delta^{floor(\log_\Delta l/t_x)}$, similarly for l_y and l_z; and $uc_x = t_x * \Delta^{ceil(\log_\Delta u/t_x)}$, similarly for uc_y and uc_z.

Choosing step sizes

The step size essentially is the inter-sample-interval for the discrete set of parameters chosen for exhaustive searches. To maximize the chances of an exhaustive search that uses a discrete set of parameter values to approximately find the global minima, the step size must be chosen so that the the difference in spatial dissimilarity corresponding to adjacent parameter values is small. Differences in spatial dissimilarity corresponding to adjacent parameter values depend on the voxel size used to discretize the volumes of the morphologies. For example, in most cases, when translations smaller than the voxel size are applied, only a small fraction of the set of voxels representing the morphology change and hence the spatial dissimilarity between the morphologies also changes by a small amount. Therefore, we choose a step size depending on the voxel size being used.

For a voxel size g, the step size Δ for discretizing the parameter space is chosen as follows:

- **Translation:** $\Delta = g$
- **Rotation:** $\Delta = \frac{g}{maxdist}$
- **Scaling:** $\Delta = \frac{maxdist}{maxdist - g}$

where $maxdist$ is the distance of the farthest point of the morphology from its centroid.

Similar to translation, the step size for rotation (scaling) is chosen as the angle (scale) which moves a point by a distance of one voxel size. Since this angle (scale) depends on the distance of the point being moved from the centroid of the morphology, we choose the smallest of such angles (scales), the one which corresponds to the farthest point of the morphology from its centroid.

Error in estimation

Using an exhaustive search over a discrete set of transforms to find a transform that minimizes spatial dissimilarity between morphologies inherently introduces an uncertainty since it does not search through all possible transforms, but only a subset of them. However, with exhaustive searches that use equally spaced set of transforms, this uncertainty is bounded above by the spacing between transforms and thus can be made very small by choosing a closely spaced set of transforms. Additionally, the estimate could contain an uncertainty that is due to volume discretization of the morphologies. To illustrate this effect, consider the case where two morphologies have just one point each. Since Reg-MaxS calculates spatial dissimilarity based on voxel overlap, any transform that brings the two points within the same voxel would be a solution. For cases where the points are near voxel boundaries, an exhaustive search over a discrete set of equally spaced transformations would prefer a transform that brings the two points into the same voxel over a transform that doesn't bring them into the same voxel but brings them closer. When morphologies have more points, such cases arise more often and this uncertainty becomes more prominent when working with experimentally acquired morphologies with hundreds or thousands of points. Thus Reg-MaxS uses mainly the uncertainty in an estimate caused by volume discretization to determine the plausible region of transforms for the next exhaustive search.

Further, for rotational and scaling differences, points at different distances from the center will have different estimation uncertainties. However, farther points dominate the estimation procedure as they are more sensitive to transformations. Hence, we use the farthest point when calculating estimation uncertainty.

Translation:

Since we apply translations along orthogonal axes, the errors can be calculated independently. This implies that, for each parameter, we need to consider the effect of discretization only along its corresponding axis. The two extreme cases of maximum positive and negative errors occur when discretization causes the separation between two points to maximally decrease and increase respectively. These are illustrated in Fig. 1(a) for the case where each morphology has just one point each. These two points are separated along an axis (X, Y or Z) by a distance of l and the voxel size used for discretization is g. In this case maximal errors in estimating translation parameters can be calculated as follows:.

Translation difference between discretized morphologies	$\delta_t = l$
Smallest possible difference before discretization	$\delta_{min} = l - g$
$\Rightarrow$ Maximum negative error	$\delta_- = g$
Largest possible difference before discretization	$\delta_{max} = l + g$
$\Rightarrow$ Maximum positive error	$\delta_+ = g$

Thus we can expect an error of plus-or-minus one voxel size when estimating translation parameters.

Scaling:

Consider subfigure (a) in S4_Figure with similar notations as above. Let the points be at a large distance D from the centroid of the morphology. For a given value of l, we have:

$$\text{Scaling difference between discretized morphologies} \quad \delta_s = \frac{l+D}{D}$$

$$\text{Smallest possible difference before discretization} \quad \delta_{min} = \frac{l+D-0.5g}{D+0.5g}$$

$$\Rightarrow \text{Maximum negative error} \quad \delta_-(l) = \frac{(l+D)(D+0.5g)}{D(l+D-0.5g)} = \left(\frac{D+0.5g}{D}\right)\left(1+\frac{0.5g}{l+D-0.5g}\right)$$

$$\text{Largest possible difference before discretization} \quad \delta_{max} = \frac{l+D+0.5g}{D-0.5g}$$

$$\Rightarrow \text{Maximum positive error} \quad \delta_+(l) = \frac{(l+D+0.5g)D}{(D-0.5g)(l+D)} = \left(\frac{D}{D-0.5g}\right)\left(1+\frac{0.5g}{l+D}\right)$$

Thus, for a given value of D, the estimation errors decrease with increasing l. Hence, to calculate maximum possible error, we use the least possible value of l which is g. Thus, substituting, we have:

$$\text{Maximum positive error} \quad \delta_+ = \frac{D+g}{D}$$

$$\text{Maximum negative error} \quad \delta_- = \frac{(D+1.5g)D}{(D-0.5g)(D+g)}$$

Rotation:

Consider subfigure (b) in S4_Figure, with similar notations as above. Since rotation about an axis causes a point to move in a 2D plane, we need to consider the effect of discretization along two axes. The greatest possible increase and decrease of separation between two points will happen when they are separated along the diagonal of a voxel face as shown in subfigure (b) in S4_Figure.

We calculate error for a point farthest from the centroid of the morphology, which is at a distance D. Assuming $l \ll D$, since $\sin^{-1} x \sim x$ for small x and for a voxel size of g, we have,

$$\text{Rotation difference between discretized morphologies} \quad \delta_r = \frac{l}{D}$$

$$\text{Smallest possible difference before discretization} \quad \delta_{min} = \frac{l-g\sqrt{2}}{D}$$

$$\Rightarrow \text{Maximum negative error} \quad \delta_- = \frac{g\sqrt{2}}{D}$$

$$\text{Largest possible difference before discretization} \quad \delta_{max} = \frac{l+g\sqrt{2}}{D}$$

$$\Rightarrow \text{Maximum positive error} \quad \delta_+ = \frac{g\sqrt{2}}{D}$$

Significance of algorithm parameters and how to set them

Bounds for transformation parameters:

As part of Reg-MaxS, we estimate rotation, translation or scaling errors between morphologies by estimating corresponding parameters. Since the algorithm uses exhaustive search over the parameter space at successively decreasing voxel sizes, it needs lower and upper bounds to sample the parameter space.

These bounds define ranges in which these parameters are expected to be found. If the actual solution is outside these bounds, the solution given by Reg-Max-S might be a local minimum within the specified parameter bounds or at the bound closer to the actual solution, which could be sub-optimal. Setting

these parameter bounds appropriately, either using good contextual estimates or by experimentation, can significantly improve the spatial overlap of the final solution reached.

The runtime of exhaustive search, which is the largest component of Reg-MaxS runtime, increases roughly linearly with the number of sample points of the parameter space searched. The total number of parameter sample points searched is the product of the number of sample points along each axis. For the highest voxel size, these are determined by the input bound parameters and the step size. For exhaustive searches at lower voxel sizes, this is fixed as the bounds are chosen depending on the current estimate error. Thus, these bound parameters affect the runtime of Reg-MaxS directly.

Since we expect hemispheric reflections to be taken care of beforehand, multiple morphologies of a stereotypic neuron obtained using similar imaging and reconstruction methods are not expected to have large translational, rotational or scaling differences.

Tolerance for transformation parameters:

Tolerances need to be set for each of the translational, rotational and scaling parameters. They need to be set to values lower than their corresponding step sizes at the lowest voxel size. After successively refining parameter estimates at several decreasing voxel, a final exhaustive search is run over the parameter space using these tolerances as the step size. Note that these do not set the final accuracy of the estimate. They are set by parameter step sizes calculated at the lowest voxel size.

Sequence of Voxel Sizes:

The algorithm requires a sequence of voxel sizes which are used to discretize morphologies and are the most important set of parameters for Reg-MaxS. The lowest voxel size influences the final accuracy of parameter estimates. Hence the lowest voxel size must be chosen small, enough to preserve the features of the arbor being studied but large enough to average out the inter-specimen variability in dendrites.

For exhaustive searches at all voxel sizes other than the highest, the bounds of each parameter are chosen to be directly proportional to the next highest (previous) voxel size, while the step size is proportional to the current voxel size (See "Choosing step sizes"). Thus the number of sample points of the parameter space to search at each voxel size is directly proportional to the cube of the ratio of successive voxel sizes. Thus this ratio affects the runtime of Reg-MaxS and must be set to a small value. However, if this ratio is too small, parameter refinement at each voxel size is small and the algorithm would require refinements at several voxel sizes to achieve enough accuracy.

Initial Reference:

The choice of the initial reference has a major impact on the outcome of the both the PCA-based method and Reg-MaxS-N. This initial reference has to be the best representative morphology of the neuron in the set being studied for the algorithms to perform the best. It must have the most complete arborization among the morphologies being registered and also the least amount of artifacts.

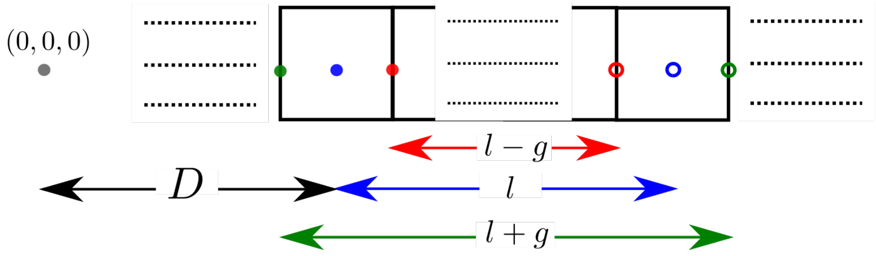

(a)

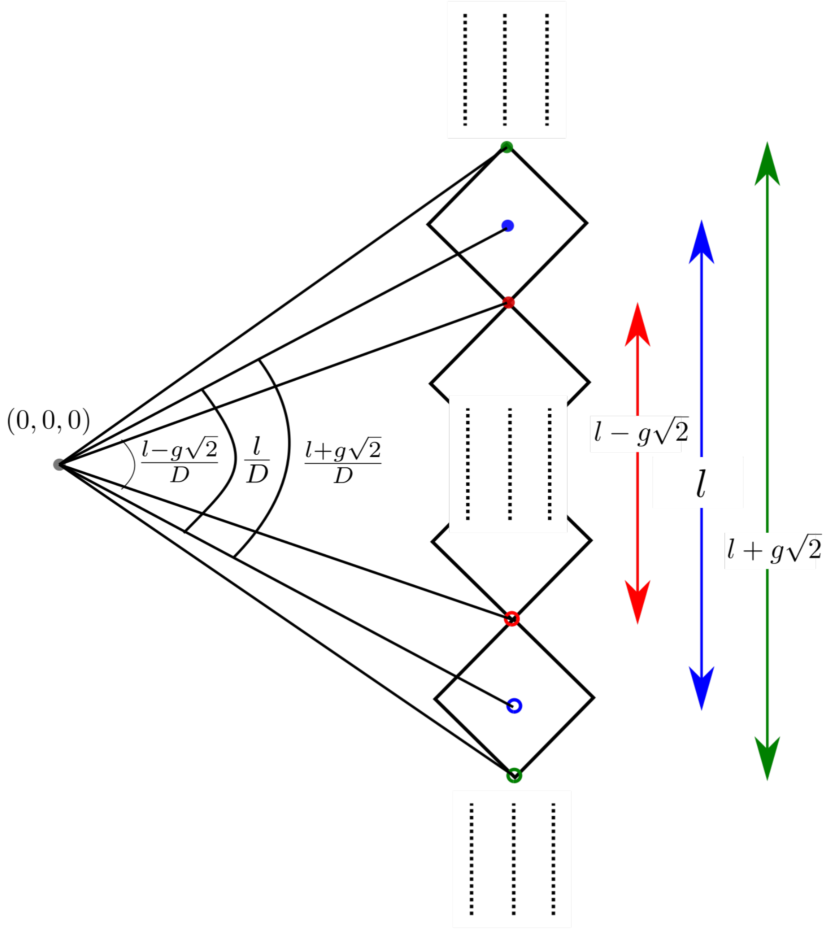

(b)

Figure 1: **Illustrating errors in estimating best transformation parameters due to volume discretization** Squares indicate representative voxels used to discretize the morphologies. Points in blue are discretized points, red and green are the points before discretization for the cases that lead to maximum negative and maximum positive errors, respectively. (a) For the case of estimating translation and scaling parameters. The two discretized points of the morphologies are separated along an axis (X, Y or Z) by a distance of l. (b) For the case of estimating rotational parameters. This is for the case where the two points are at an approximate large distance of D from the centroid of the morphology and are separated diagonally along a voxel by a distance of l.

Algorithms

Algorithm 1: Multi-scale estimation algorithm

Data: the two morphologies, a set of $n(> 0)$ monotonically decreasing voxel sizes $[g_1, g_2, \cdots, g_n]$, bounds for each parameter being estimated and a minimum tolerance(Δ_{min})

Result: transform parameter estimates

$i \leftarrow 1$

$currentBounds \leftarrow$ initial parameter bounds

foreach g_i *in* $[g_1, g_2, \cdots, g_n]$ **do**

    $stepSize \leftarrow$ step size at g_i

    Estimate transform parameters which maximize spatial overlap with $currentBounds$, $stepSize$ and g_i

    Calculate maximum positive ($maxerr_+$) and negative ($maxerr_-$) errors in the transform parameter estimates

    if *Translation or rotation estimation* **then**

        $currentBounds \leftarrow [estimate - maxerr_-, estimate + maxerr_+]$

    end

    if *Scaling estimation* **then**

        $currentBounds \leftarrow [estimate/maxerr_-, estimate * maxerr_+]$

    end

end

if $stepSize > \Delta_{min}$ **then**

    Estimate transform parameters which maximize spatial overlap with $currentBounds$, Δ_{min} and g_n

end

Algorithm 2: Reg-MaxS

```
Data: Reference and test morphologies, algorithm parameters
Result: Test morphology registered to the reference
Register the test approximately to the reference
currentMorph ← approximately registered morphology
scaleDone ← False
while scaleDone == False do
    done ← False
    while done == False do
        Estimate rotation and translation errors between currentMorph and reference morphology
          using multi-scale method
        Select the transform which, upon application, results in lowest spatial dissimilarity
        Apply this transform to currentMorph to generate tempMorph
        currentMorph ← tempMorph
        params ← parameters of the transform chosen above
        if tempMorph has higher spatial dissimilarity compared to currentMorph then
            done ← True
        else if tempMorph has same spatial dissimilarity as currentMorph then
            if any parameter has reached it's resolution limit then
                done ← True
            end
    end
    Estimate scaling error between currentMorph and reference morphology
    Apply this scaling to currentMorph to generate tempMorp
    currentMorph ← tempMorph
    params ← parameters of the scaling applied
    if tempMorph has higher spatial dissimilarity compared to currentMorph then
        scaleDone ← True
    else if tempMorph has same spatial dissimilarity as currentMorph then
        if any parameter has reached it's resolution limit then
            done ← True
        end
end
Among all the iterations above, chose the iteration with lowest spatial dissimilarity as final solution
```

Algorithm 3: Reg-MaxS-N

Data: Group of morphologies, algorithm parameters

Result: Morphologies registered to each other

Notation: $ref(iterNo)$ →the reference at the end of iteration $iterNo$

Notation: $morph(iterNo, morphInd)$ →morphology with index $morphInd$ at the end of iteration $iterNo$

Notation: $notDone(morphInd)$ →Indicates whether any further improvements are possible for morphology with index $morphInd$

foreach $morphInd$ **do**
    $notDone(morphInd) \leftarrow True$
end
$iterNo \leftarrow 0$
$ref(-1) \leftarrow$ one of the morphologies
foreach $morphInd$ **do**
    $morph(-1, morphInd) \leftarrow$ approximate registration of morphology with index $morphInd$
end
while $any(notDone(morphInd) == True)$ **do**
    foreach $morphInd$ **do**
        $notDone(morphInd) \leftarrow True$
        Register $morph(iterNo - 1, morphInd)$ to reference $ref(iterNo - 1)$ to generate $morph(iterNo, morphInd)$
        if *registration is rejected* **then**
            $morph(iterNo, morphInd) \leftarrow morph(iterNo - 1, morphInd)$
            $notDone(morphInd) \leftarrow False$
        end
        Calculate the total scale applied to $morph(iterNo - 1, morphInd)$ in the iteration and adjust the scale bounds for next iteration
    end
    $ref(iterNo) \leftarrow$ union of all $morph(iterNo, .)$
end
Among all the iterations above, chose the iteration with the lowest occupancy based dissimilarity measure at the lowest voxel size to be the final solution
Transform all morphologies with a transform that inverse projects the final form of the initial reference to its original form.
