# Supplementary material for: Spatial registration of neuron morphologies based on maximization of volume overlap
Source: BMC Bioinformatics. 2018 Apr 18;19:143. doi: 10.1186/s12859-018-2136-z (PMC5907365; doi:10.1186/s12859-018-2136-z)
Supplement: Supplementary file 3 — Performance comparison of Reg-MaxS-N and other methods for different initial references plotted separately for each group of morphologies. (PDF 593 kb) [file 12859_2018_2136_MOESM3_ESM.pdf]

Additional File 3:
Comparing algorithm performances for different initial references

Part of "Spatial registration of neuron morphologies based on maximization of volume overlap"

Ajayrama Kumaraswamy[1], Kazuki Kai[2], Hiroyuki Ai[2], Hidetoshi Ikeno[3], Thomas Wachtler[1]

1. Department of Biology II, Ludwig-Maximilians Universität München, Martinsried, Germany
2. Department of Earth System Science, Fukuoka University, Fukuoka, Japan
3. School of Human Science and Environment, University of Hyogo, Himeji, Japan

LCInt

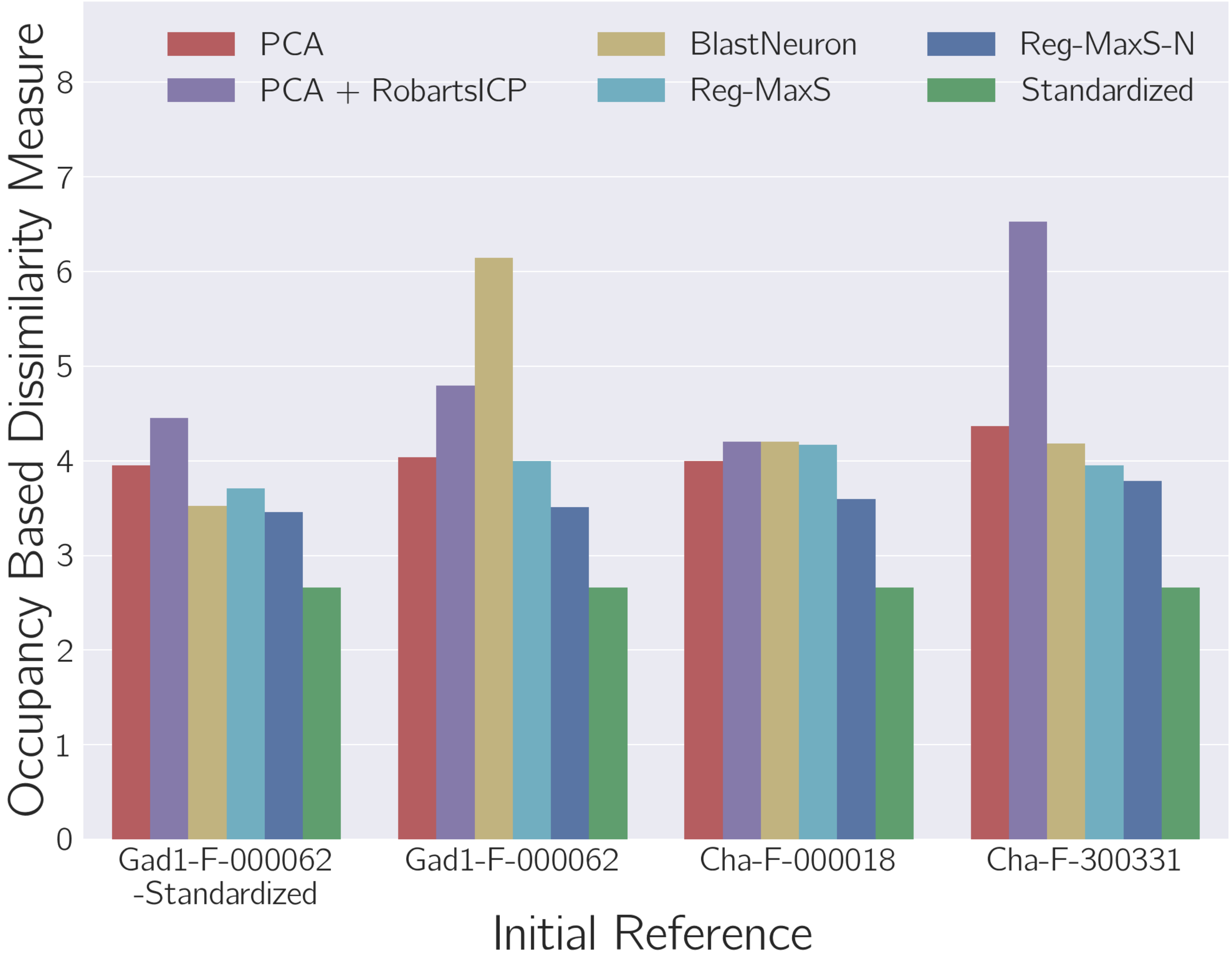

Figure A31: Comparing the performance of six registration algorithms for LCInt group of morphologies, for four different initial references.

ALPN

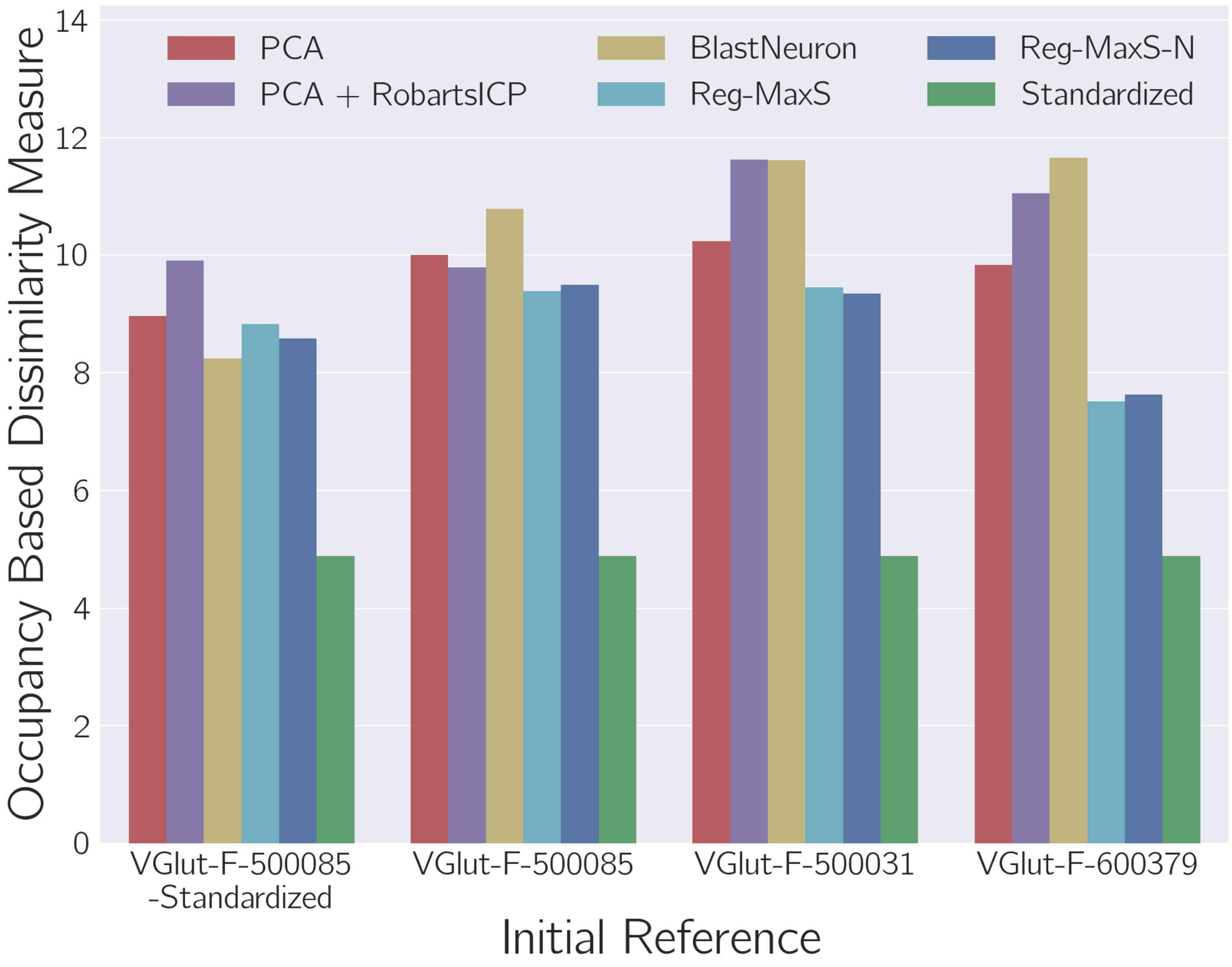

Figure A32: Comparing the performance of six registration algorithms for ALPN group of morphologies, for four different initial references.

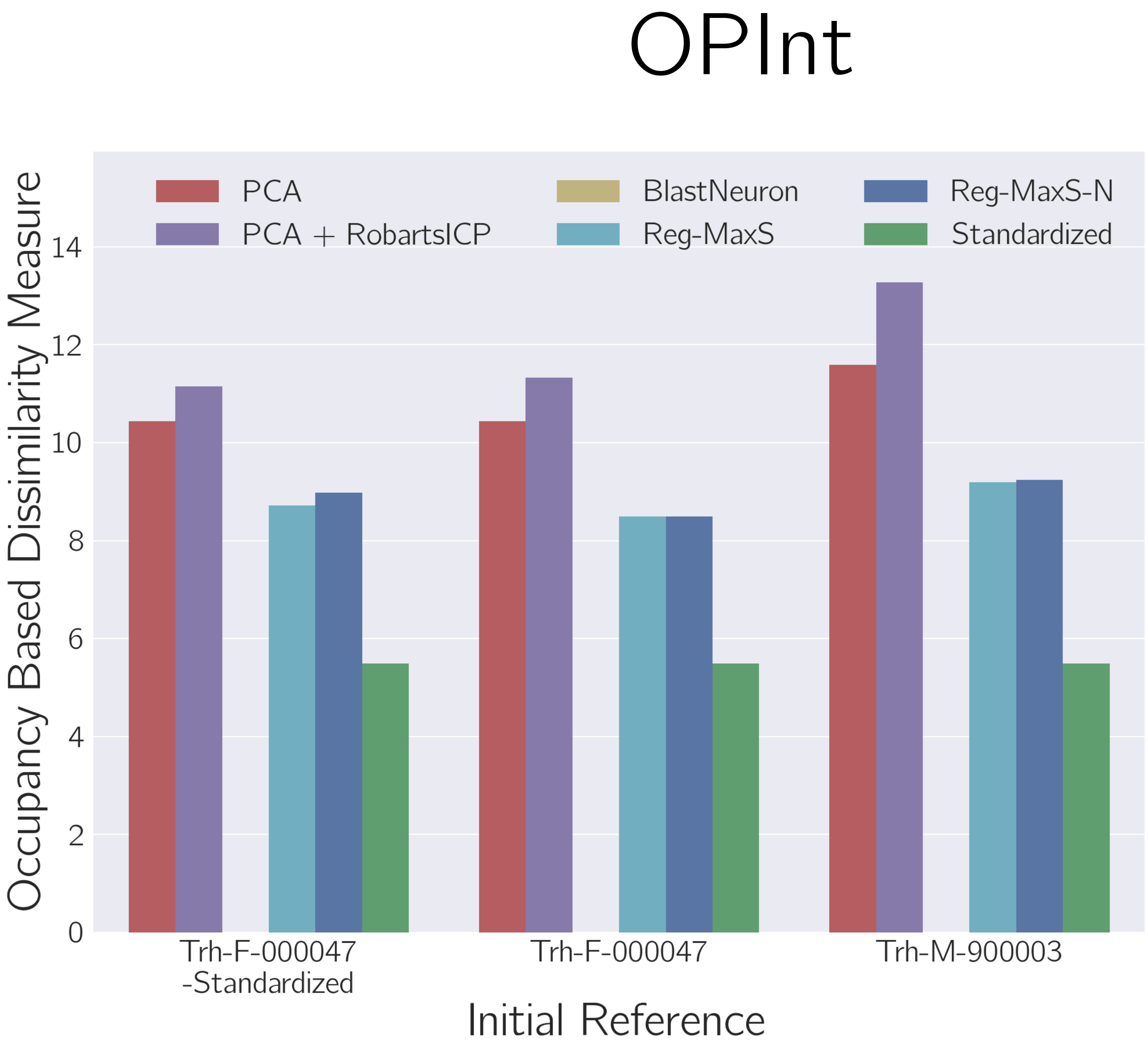

Figure A33: Comparing the performance of six registration algorithms for OPInt group of morphologies, for three different initial references. BlastNeuron performances are not shown as the program provided by the authors stopped after an internal time limit of 30 minutes and produced no results.

AA1

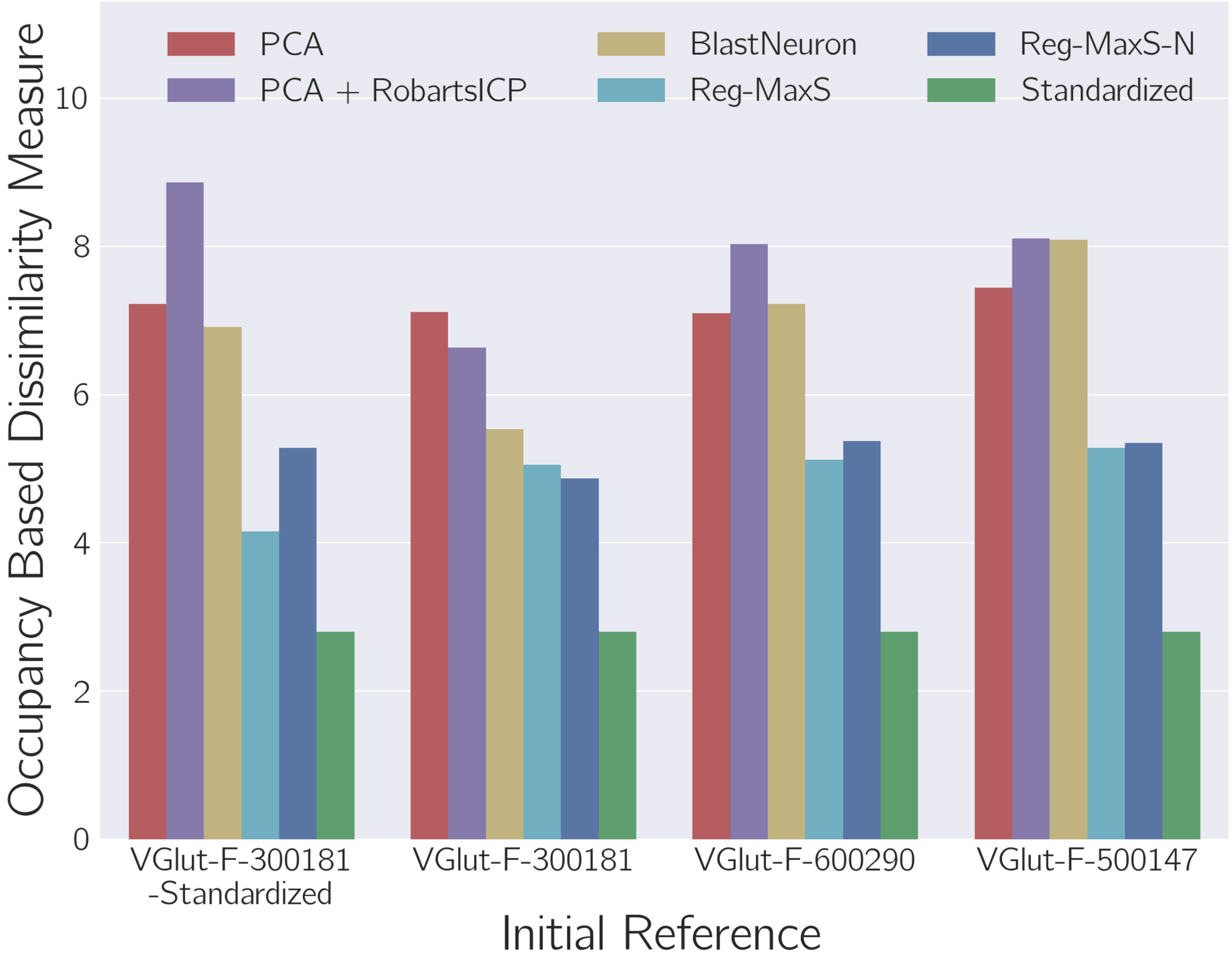

Figure A34: Comparing the performance of six registration algorithms for AA1 group of morphologies, for four different initial references.

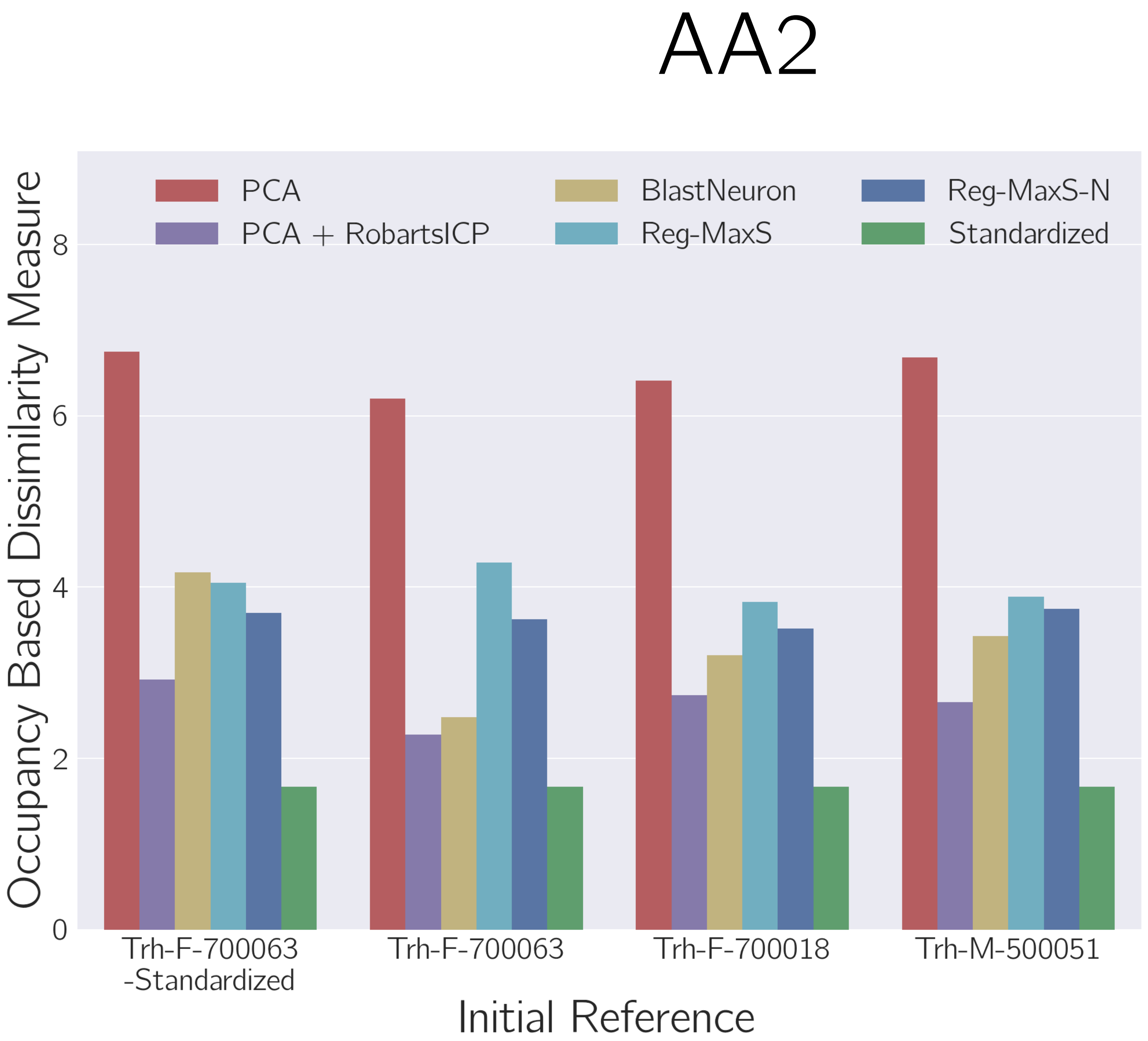

Figure A35: Comparing the performance of six registration algorithms for AA2 group of morphologies, for four different initial references.
