# Supplementary material for: Spatial registration of neuron morphologies based on maximization of volume overlap
Source: BMC Bioinformatics. 2018 Apr 18;19:143. doi: 10.1186/s12859-018-2136-z (PMC5907365; doi:10.1186/s12859-018-2136-z)
Supplement: Supplementary file 4 — Tests Results of Reg-MaxS and Reg-MaxS-N with smallest voxel size set to 5μm. (PDF 181 kb) [file 12859_2018_2136_MOESM4_ESM.pdf]

Additional File 4: Tests Results of Reg-MaxS and Reg-MaxS-N with smallest voxel size set to $5\mu m$

as part of "Spatial registration of neuron morphologies based on maximization of volume overlap"

Ajayrama Kumaraswamy*[1], Kazuki Kai[2], Hiroyuki Ai[2], Hidetoshi Ikeno[3], and Thomas Wachtler†[1]

[1]Dept. Biologie II, Ludwig-Maximilians-Universität München
[2]Department of Earth System Science, Fukuoka University, Fukuoka, Japan
[3]School of Human Science and Environment, University of Hyogo, Himeji, Japan

Testing Reg-MaxS

Testing Reg-MaxS with synthetic morphologies

Tests of Reg-MaxS using 1000 noiseless morphologies were repeated with the smallest voxel size set to $5\mu m$. When point wise distance statistics were calculated for each test registration across SWC points, 322 of 1000 tests (32.2%) had final distances that were significantly smaller than the smallest voxel size (n=1290, Signs Test, 1% significance level). This is down from 67.4% for tests where smallest voxel size was set to $10\mu m$. When point wise distance statistics were calculated for each SWC point across test registrations, 26 of 1290 SWC points (2%) had final distances that were significantly smaller than the smallest voxel size (n=1000, Signs Test, 1% significance level). This is down from 99.76% for tests where smallest voxel size was $10\mu m$. It's important to note that these synthetic morphologies were about 10 times larger than the other test morphologies and had fewer features at scales smaller than $10\mu m$, which is the main reason for the drop in performance seen at $5\mu m$.

Testing Reg-MaxS with real reconstructions

Tests of Reg-MaxS that co-registered non-standard versions of LCInt morphologies to corresponding standardized versions were repeated with the value of the smallest voxel size set to 5um. Reg-MaxS performed well on all morphologies, with significant number of nearest point pairs closer than the smallest voxel size ($117 \leq n \leq 276$, Signs test, 1% significance level). Final distances between nearest neighbors of result and reference morphologies ($1.79 \pm 1.32\mu m$) were lower compared to those when the smallest voxel size was $10\mu m(5.51 \pm 4.49\mu m)$, indicating finer alignment in the former case. It's important to note that these test morphologies were smaller in size and had more features at spatial scales lesser than $10\mu m$ compared to synthetic test morphologies above, which is the main reason for improved Reg-MaxS performance for real morphologies.

*ajayramak@bio.lmu.de
†wachtler@bio.lmu.de

Testing Reg-MaxS-N

Reg-MaxS and Reg-MaxS-N were reapplied with the smallest voxel size set to $5\mu m$ to co-register four groups of morphologies from Drosophila Melanogaster that were used in the main manuscript. The registrations were repeated with four different initial references for every group, similar to tests reported in the main manuscript. Fig A41 compares the performances of Reg-MaxS and Reg-MaxS-N applied with two values for smallest voxel size to the performances of BlastNeuron and brain atlas based method quantified by occupancy based similarity measure defined in the main manuscript. The performances of Reg-MaxS and Reg-MaxS-N did not change remarkably when the smallest voxel size was changed from $10\mu m$ to $5\mu m$. Performance increased or decreased depending on the group of morphologies and the initial reference used. Note that this performance quantifies the spatial alignment of a group of morphologies and is different from the performances discussed in the previous sections, which quantifies the reliability of spatial alignment of pairs of morphologies across transformation differences.

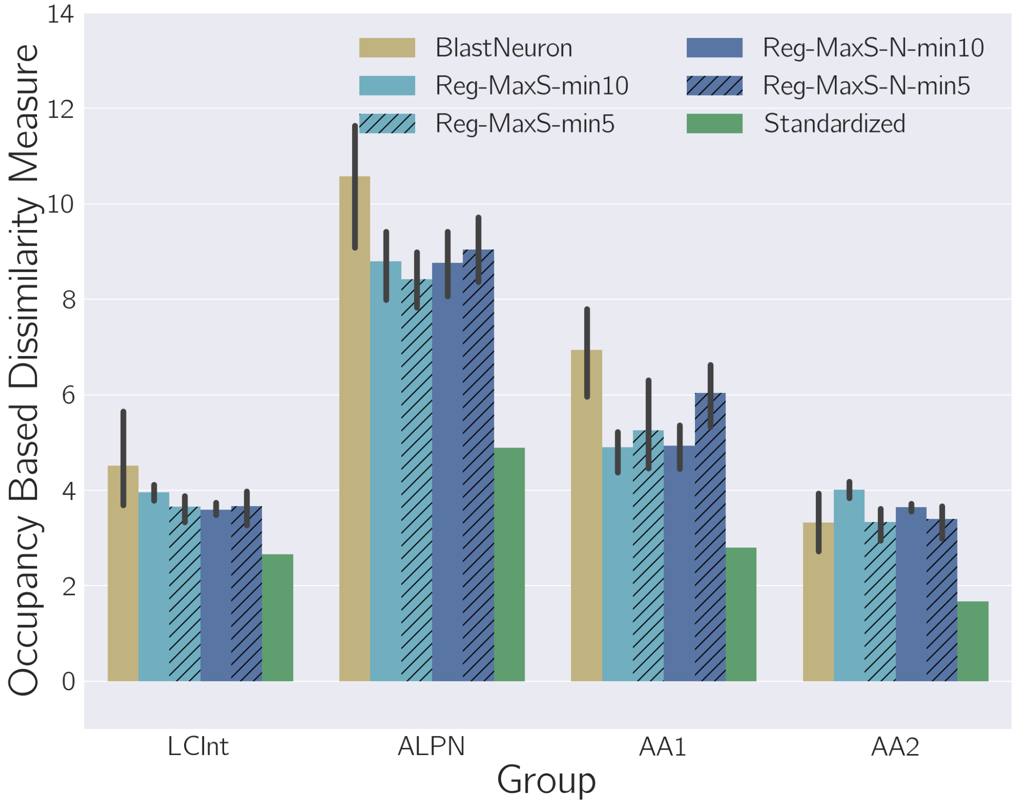

Figure A41: Comparing the performance of Reg-MaxS and Reg-MaxS-N with BlastNeuron and standard brain based method for different values of smallest voxel size. Performances did not show any remarkable changes when smallet voxel size was changed from $10\mu m$ to $5\mu m$
